# Enhancement of 5-Fluorouracil Drug Delivery in a Graphene Oxide Containing Electrospun Chitosan/Polyvinylpyrrolidone Construct

**DOI:** 10.3390/ma17215300

**Published:** 2024-10-31

**Authors:** Jamie J. Grant, Suresh C. Pillai, Tatiana S. Perova, Barry Brennan, Steven J. Hinder, Marion McAfee, Sarah Hehir, Ailish Breen

**Affiliations:** 1 Nanotechnology and Bio-Engineering Research Group, Health and Biomedical (HEAL) Strategic Research Centre, Atlantic Technological University, Ash Lane, F91 YW50 Sligo, Ireland; suresh.pillai@atu.ie (S.C.P.); barry.brennan@atu.ie (B.B.); sarah.hehir@atu.ie (S.H.); ailish.breen@atu.ie (A.B.); 2 Department of Electronic and Electrical Engineering, Trinity College Dublin, The University of Dublin, College Green, D02 PN40 Dublin, Ireland; perovat@tcd.ie; 3 The Surface Analysis Laboratory, University of Surrey, Stag Hill, Guildford GU2 7XH, UK; s.hinder@surrey.ac.uk; 4 Centre for Mathematical Modelling and Intelligent Systems for Health and Environment (MISHE), Atlantic Technological University Sligo, Ash Lane, F91 YW50 Sligo, Ireland; marion.mcafee@atu.ie; 5 I-Form, The SFI Research Centre for Advanced Manufacturing, Atlantic Technological University Sligo, Ash Lane, F91 YW50 Sligo, Ireland

**Keywords:** chitosan, electrospinning, PVP, graphene oxide, cytotoxicity, Raman, 5-Fu, FTIR, biomedical engineering

## Abstract

Electrospun nanofibrous mats, consisting of chitosan (CS) and polyvinylpyrrolidone (PVP), were constructed with the addition of graphene oxide (GO) for enhancement of delivery of the 5-Fluorouracil (5-Fu) chemotherapy drug. Upon studying the range of GO concentrations in CS/PVP, the concentration of 0.2% *w*/*v* GO was chosen for inclusion in the drug delivery model. SEM showed bead-free, homogenous fibres within this construct. This construct also proved to be non-toxic to CaCo-2 cells over 24 and 48 h exposure. The construction of a drug delivery vehicle whereby 5-Fu was loaded with and without GO in various concentrations showed several interesting findings. The presence of CS/PVP was revealed through XPS, FTIR and Raman spectroscopies. FTIR was also imperative for the analysis of 5-Fu while Raman exclusively highlighted the presence of GO in the samples. In particular, a detailed analysis of the IR spectra recorded using two FTIR spectrometers, several options for determining the concentration of 5-Fu in composite fibre systems CS/PVP/5-Fu and GO/CS/PVP/5-Fu were demonstrated. By analysis of Raman spectra in the region of D and G bands, a linear dependence of ratios of integrated intensities of *A_D_* and *A_G_* on the intensity of host polymer band at 1425 cm^−1^ vs. GO content was found. Both methods, therefore, can be used for monitoring of GO content and 5-Fu release in studied complex systems. After incorporating the chemotherapy drug 5-Fu into the constructs, cell viability studies were also performed. This study demonstrated that GO/CS/PVP/5-Fu constructs have potential in chemotherapy drug delivery systems.

## 1. Introduction

Due to its interesting material properties, there has been increasing study and use of graphene oxide (GO) as a potential material in biomedical science [1,2], including in anti-cancer applications [3,4]. GO is a two-dimensional anatomically thin carbon-based material, consisting of sp^2^ carbon atoms structured in a honeycomb lattice format [5]. GO is hydrophilic in nature due to an abundance of oxygen-based functional groups. Its large surface area makes it a potential candidate for drug delivery purposes [6]. It has been reported that GO may readily load drugs with high efficiency while improving hydrogen bonding between the drugs and the nanocarrier [7]. One such drug is 5-Fluorouracil (5-Fu). 5-Fu is regarded as one of the most widely used antimetabolite chemotherapeutic agents in recent decades [8]. It is the mainstay treatment for early-stage colon cancer [9].

There have been mixed reports on the toxicity of GO. While most findings have shown that the material is biocompatible and non-toxic [10,11], it has also been shown that at high concentrations, GO may provoke a loss in biocompatibility [12,13]. If we wish to create a drug delivery vehicle incorporating GO, it is important to ensure the resultant material uses inherently non-toxic, safe co-constituents to form a composite.

Electrospinning is a dynamic method of fibre production, able to produce fibres ranging from the sub-micron to the nano-scale and which is often used in drug delivery mechanisms [14,15]. The principle of electrospinning involves the subjection of a loaded polymer solution, via needle tip, to a high-voltage power supply [16]. The applied voltage overcomes the surface tension of the polymer and produces a “Taylor Cone”, which results in an electrically charged jet emitted from the needle tip [17]. This study aims to combine two biocompatible polymers, chitosan (CS) and polyvinylpyrrolidone (PVP), to act as a backbone for the delivery of 5-Fu, whereby GO may act as the drug carrier in the composite.

CS, the second most abundant biopolymer in nature, possesses favourable bio-credentials such as its biocompatibility, biodegradability, and non-toxic and antibacterial properties [18]. However, CS has poor processability due to its limited solubility [19]. Because of this, CS has been historically co-blended with other similar bio-enhanced materials to improve solubility [20,21]. PVP is one such material. This hydrophilic polymer is often used in biomedical fields due to its low toxicity, biocompatibility, and biodegradability [22].

There have been some previously reported models that have incorporated 5-Fu and/or GO in electrospun scaffolds for drug delivery purposes. Jouybari et al. have successfully created a drug delivery system where 5-Fu was successfully released from a CS/PVA electrospun construct, targeting MCF-7 breast cancer cells [23]. Grant et al. utilized a CS/PVP/5-Fu vehicle to target A549 lung cancer cells, showing a 10 mg/mL loading to be most successful at killing cells [14]. Lee et al. targeted YD-10B tongue cancer cells using 5-Fu immobilized hyaluronic acid hydrogen arrays on an electrospun bilayer membrane [24]. All of these reported an efficient, sustainable-release model which could have potential in biomedical applications. Recent delivery methods of 5-Fu include Soy Lecithin Vesicles [25], Dextran-based nanoparticles [26], gold-polydopamine nanocomplexes [27], and zein/sericin nanoblends [28].

5-Fu has a poor pharmacokinetic profile and can cause severe health problems due to its limited therapeutic window [29]. Until now there has been no assessment of the potential for 5-Fu to be delivered via GO/CS/PVP electrospun scaffolds, targeting colon cancer cells (CaCo-2). This scaffold may act as a safer vehicle for 5-Fu delivery, removing the adverse side effects (fever, nausea, vomiting, anemia, rashes, etc.) [30] associated with intravenous administration. However, it is initially critical to study a range of GO concentrations to ensure proper biocompatibility before drug inclusion. Once this is established, it is then possible to create 5-Fu-loaded GO/CS/PVP scaffolds to allow assessment of the drug loading/release and efficiency of cellular kill regarding human colon cancer cells with the end goal of creating an effective anti-cancer drug delivery vehicle.

## 2. Materials and Methods

### 2.1. Materials

Low-Molecular-Weight CS (mol wt 50,000–190,000) (75–85% deacetylated) (chosen due to reported viscosity issues when Electrospinning High-Molecular-Weight CS [31]) was acquired. Trifluoro Acetic Acid (TFA) (99%) and (Glacial Acetic Acid) GAA (≥99.85%) were purchased from Sigma-Aldrich (St. Louis, MO, USA). High-Molecular-Weight PVP (mol wt 1,300,000) (selected as this Molecular Weight is generally the easiest PVP to Electrospin [32]) was purchased from VWR. Graphene Oxide was purchased from Sigma-Aldrich in sheet form. In addition, 4–10% edge-oxidized, DMEM medium, penicillin–streptomycin solution, trypsin (pure from beef pancreas), and non-essential amino acids (NEAAs) were purchased from Fisher Scientific (Waltham, MA, USA). Phosphate Buffer Saline (ultra-pure grade), Fetal Bovine Serum (Ireland Origin), and WST-8 Cell Proliferation Assay Kit were purchased from VWR. CaCo-2 cells were donated by ATU Sligo from their cell bank store. The cells were purchased from Sigma-Aldrich (lot number: 18H002), supplied by European Collection of Authentic Cell Cultures (ECACC), ECACC 86010202.

### 2.2. Varying GO Concentration in CS/PVP Electrospun Composites

#### 2.2.1. Solution Preparation

Subsequently, 4% CS, 6% PVP (% *w*/*v*) were dissolved in TFA:GAA in a 9:1 ratio in five separate vessels (to create a 40:60 CS/PVP ratio). These quantities were chosen as per previous studies by Grant et al., 2021 [14]. Caution was exercised when using harsh solvents such as TFA. Solvent evaporation during electrospinning requires good ventilation. Samples were left to stir at room temperature for approximately 24 h. Following dissolution, samples were prepared as follows; (a) CS/PVP only (b) 0.1% *w*/*v* GO added (c) 0.2% *w*/*v* GO added (d) 0.5% *w*/*v* GO added and (e) 0.7% *w*/*v* GO added.

#### 2.2.2. Electrospinning Procedure

Samples (a) to (e) were electrospun using a SprayBase™ electrospinning kit (Avectas, Dublin, Ireland). The following instrumental parameters were used: 0.2 mL/h flow rate, 10 cm tip-collector gap, 10 kV DC. Samples were electrospun for approximately six hours on aluminium foil and carefully removed using forceps.

### 2.3. Study of 0.2 w/v% GO in CS/PVP Electrospun Composites with Varying Concentrations of 5-Fu

#### 2.3.1. Solution Preparation Containing 5-Fu

Then, 4% CS, 6% PVP (%*w*/*v*) were dissolved in TFA:GAA (9:1) as above, in six separate vessels. Samples were then prepared as follows: (a1) 0.2% *w*/*v* GO and 1 mg/mL 5-Fu added to CS/PVP solution, (b1) 0.2% *w*/*v* GO and 5 mg/mL 5-Fu added to CS/PVP solution, (c1) 0.2% *w*/*v* GO and 10 mg/mL 5-Fu added to CS/PVP solution, (a2) 1 mg/mL 5-Fu added to CS/PVP solution, (b2) 5 mg/mL 5-Fu added to CS/PVP solution, and (c2) 10 mg/mL 5-Fu added to CS/PVP solution (see Table S1).

#### 2.3.2. Electrospinning Procedure of 5-Fu Containing Samples

Samples (a1) to (c1) and (a2) to (c2) were electrospun using a SprayBase™ electrospinning kit. The following instrumental parameters were used: 0.2 mL/h flow rate, 15 cm tip-collector gap, 15 kV DC. Samples were electrospun for approximately six hours. Following collection, samples were dried in an oven at 120 °C.

### 2.4. Characterisation

A Zeiss Gemini Ultra (Zeiss, Oberocken, Germany) was used to perform Scanning Electron Microscopy (SEM) on each sample. ImageJ software V1.54k was used to perform fibre diameter measurements, where 30 random fibres of each sample were measured. XPS analyses were performed on a ThermoFisher Scientific Instruments (East Grinstead, UK) K-Alpha+ spectrometer. XPS spectra were acquired using a monochromated Al Kα X-ray source (hν = 1486.7 eV). An X-ray spot of ~400 μm radius was employed. Survey spectra were acquired employing a pass energy of 200 eV. High-resolution core-level spectra for all elements of interest were acquired with a pass energy of 50 eV. All high-resolution spectra were charge-referenced against the C1s peak at 285 eV and against N 1s at 400 eV (depending on sample peak consistency) to correct for charging effects during acquisition. Quantitative surface chemical analyses were calculated from the high-resolution core-level spectra following the removal of a non-linear (Shirley) background using the manufacturer Avantage software Version 5, which incorporates the appropriate sensitivity factors and corrects for the electron energy analyzer transmission function. Further peak fitting and analysis were carried out using CasaXPS (version 2.3.25PR1.0) software. Peak fitting was carried out, with careful consideration of suitable peak widths, constrained biding energy shifts, and line shapes (typically Voight functions with a mixture of Gaussian (70%) and Lorentzian (30%) components for the carbon 1 s spectra) to ensure good consistency between fits for the different samples.

The infrared spectra of all investigated samples were collected using two methods, namely the grazing angle approach and diamond tip. The first measurement method was performed on a Bruker FTIR spectrometer using a GATR (Grazing Angle Attenuated Total Reflection) attachment with a Ge hemispherical ATR element in the range of 650–6500 cm^−1^ with a resolution of 4 cm^−1^ and the scan number of 250. This type of measurement hereinafter is referred to as FTIR-GATR. The problem with these measurements was the analysis of the spectra in the regions of 1500–1850 cm^−1^ and 3540–3960 cm^−1^ due to the presence of vibrational–rotational bands of water moisture in the sample compartment. The second set of FTIR spectra for the samples under study was recorded on a Perkin Elmer Spectrum 100 FTIR spectrometer using Universal Attenuated Total Reflection (UATR) accessories with an ATR diamond prism, hereinafter referred to as FTIR-UATR. The spectra were taken in the range of 300–4000 cm^−1^ with a resolution of 1 cm^−1^ and a scanning time of 3 min. An important part of these measurements was that we could record the low-frequency and high-frequency parts of the spectra with better resolution and eliminate the effect of the presence of mainly water and carbon dioxide vapors in the atmosphere.

Raman measurements were carried out in backscattering geometry using a Renishaw 1000 micro-Raman system (Renishaw plc, New Mills, UK) with a motorized positioning stage and a Leica microscope. The excitation source was a helium-neon laser with a wavelength of 633 nm and a power of <10 mW (at the laser output). The power was kept low to prevent overheating of the sample. To further reduce the power, laser filters with a transmittance of 10% of the initial laser power were used. The laser spot was focused on the sample surface (deposited on a cleaned silicon wafer) using 50× objective lenses with a short-focus working distance. Fitting of Infrared and Raman spectra was performed using Renishaw Wire 3.2 software.

### 2.5. Drug Release

#### 2.5.1. Instrumentation Set-Up

Electrospun samples were analyzed using a PerkinElmer UV/VIS Spectrometer (Lambda 35) (Perkin Elmer, Shelton, CT, USA). UV Win Lab software (https://www.perkinelmer.com/product/kit-uvwinlab-v7-4-std-software-l6100127, accessed on 1st March 2023) was programmed with a full scan set-up, starting at 700 nm and ending at 200 nm with an ordinate mode of 1 nm slit width. Scan speed was set at 480 nm/min with a data interval of 1 nm. One cycle with a cycle time of 1 s was used.

#### 2.5.2. Experimental Analysis

A standard curve consisting of free 5-Fu in PBS (pH 7.4) was created using 3.5 µg/mL, 7.5 µg/mL, 15 µg/mL, and 30 µg/mL solutions [14]. The line equation of *y* = 0.0304*x* + 0.0297 with an R^2^ value of 0.9994 was taken from the standard curve for the determination of the concentration of 5-Fu released from electrospun scaffolds (*x* denotes the *λmax* value while *y* is the 5-Fu concentration). The wavelength, *λmax*, at which a substance has its strongest photon absorption of 5-Fu, was determined at approximately 266 nm. Electrospun samples were cut into approximately 1 cm × 1 cm squares. Three samples from each mat (a2, b2 and c2) were then immersed in 4 mL of the PBS solution (pH 7.4) in a 12-well plate which was then covered in aluminium foil and placed into an incubator at 37 °C with horizontal rotating shaking (at 80 rpm). After 3, 24, and 48 h, 0.4 mL of well content was taken and added to 3.6 mL of fresh PBS (1 in 10 dilution) to read on the UV–Vis spectrometer. A total of 0.4 mL of fresh PBS was then added back into each well. The absorbances read from the instrument were multiplied by 10 to adjust for the dilution factor. The concentration of 5-Fu released from each sample was calculated using the line equation above.

### 2.6. Cell Viability Study

#### 2.6.1. Cell Culture

CaCo-2 cells were cultured with Dulbecco’s Modified Eagle Medium (DMEM) containing 10% fetal bovine serum, 1% penicillin/streptomycin and 1% non-essential amino acids (NEAA) at 37 °C with an atmosphere of 95% air and 5% CO_2_. Cells in the logarithmic stage of growth were used for analysis.

#### 2.6.2. Cell Viability Assay

Cell viability was determined using a Water-Soluble Tetrazolium-8 (WST-8) (Cayman Chemical Company, Ann Arbor, MI, USA) assay. Cells were seeded in a 96-well flat-bottomed plate at a density of 5 × 10^4^ cells/mL with 100 μL per well. They were then incubated for 24 h, and following this, exposed to electrospun constructs for an additional 24 h and 48 h (two time points). After treatment, all media were removed from the cells. A total of 100 μL of fresh medium was added to the wells along with 10 μL of the WST-8 reagent. The plate was then incubated for two hours at 37 °C in a 5% CO_2_ incubator. The plate was read at 465 nm using a microtiter plate reader. At least three replicates were performed for each test.

#### 2.6.3. Statistical Testing

ANOVA testing was performed between groups. When significancy was shown, *t*-tests, assuming unequal variance, were carried out, *n* = 3.

## 3. Results and Discussion

### 3.1. SEM Analysis

**Figure 1:**
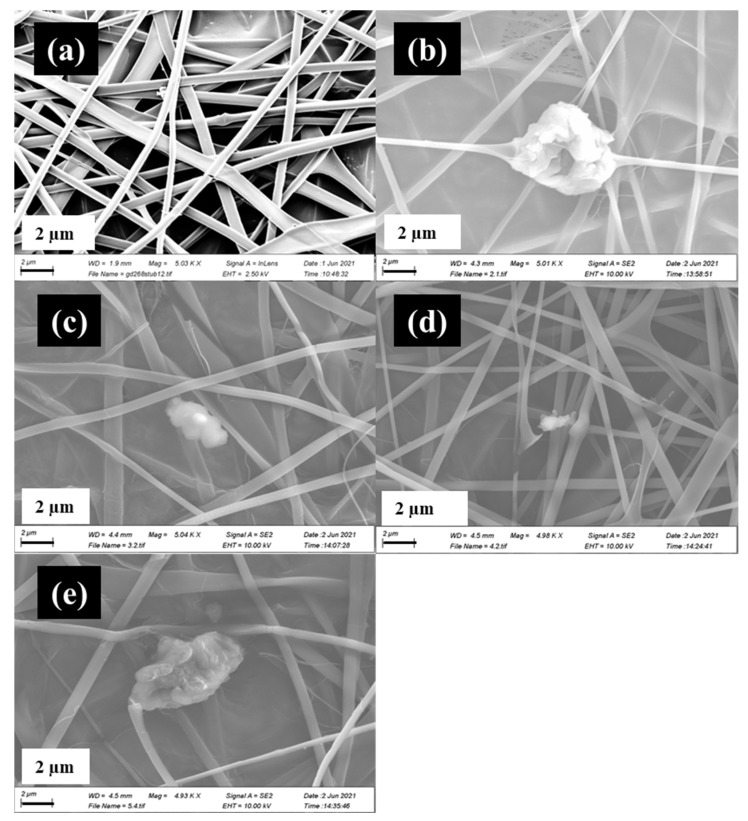
SEM images of samples (a–e) under 5 K magnification: (**a**) CS/PVP only with addition of (**b**) 0.1%*w*/*v* GO, (**c**) 0.2%*w*/*v* GO, (**d**) 0.5% *w*/*v* GO, and (**e**) 0.7% *w*/*v* GO.

As shown in Figure 1, the composites under SEM analysis exhibit homogenous random fibrous morphology in an interconnected porous structure [33]. These micrographs confirm the formation of fibres with smooth surfaces; no surface defects or porosity were detected at the micron scale. The presence of GO is also apparent, as seen in the fibrous coating as well as deposition intertwined in the fibrous matrix [34]. Average fibre diameters were calculated as follows: (a): 583 nm, (b) 534 nm, (c) 731 nm, (d) 629 nm, and (e) 738 nm (see Table S2). Sample (a) mean fibre diameter (583 nm) is similar to a previously published study by Grant et al., 2021, where it was reported that a 40% CS, 60% PVP electrospun blend had a mean fibre diameter of 569 nm [14]. Measurements of fibre diameter were subjected to one-way ANOVA testing. The mean fibre diameter of samples (a) and (b) were not statistically different (*p* = 0.3633). As the GO concentration increased, ANOVA tests showed statistical differences in fibre diameter means regarding sample (a) vs. (c) (*p* = 0.02551) and (e) (*p* = 0.00378) but not sample (d) (*p* = 0.357). Theoretically, sample (e) is expected to have the largest fibre diameter, which is seen in [33]. This may be attributed to the highest concentration of GO present in this sample. By increasing the GO concentration, the viscosity of the composite blend (pre-electrospinning) increases, thus increasing the quantity of composite interaction [33]. Another study examined electrospun tri-layered zein/PVP-GO/zein nanofibre mats for biphasic drug release [35]. PVP/GO blends produced a mean fibre diameter of 774 ± 120 nm, closely echoing the measurements taken from Table S2. Even with this, it would be expected that the samples would increase in a linear trend as the GO concentration increases, but this is not the case here.

**Figure 2:**
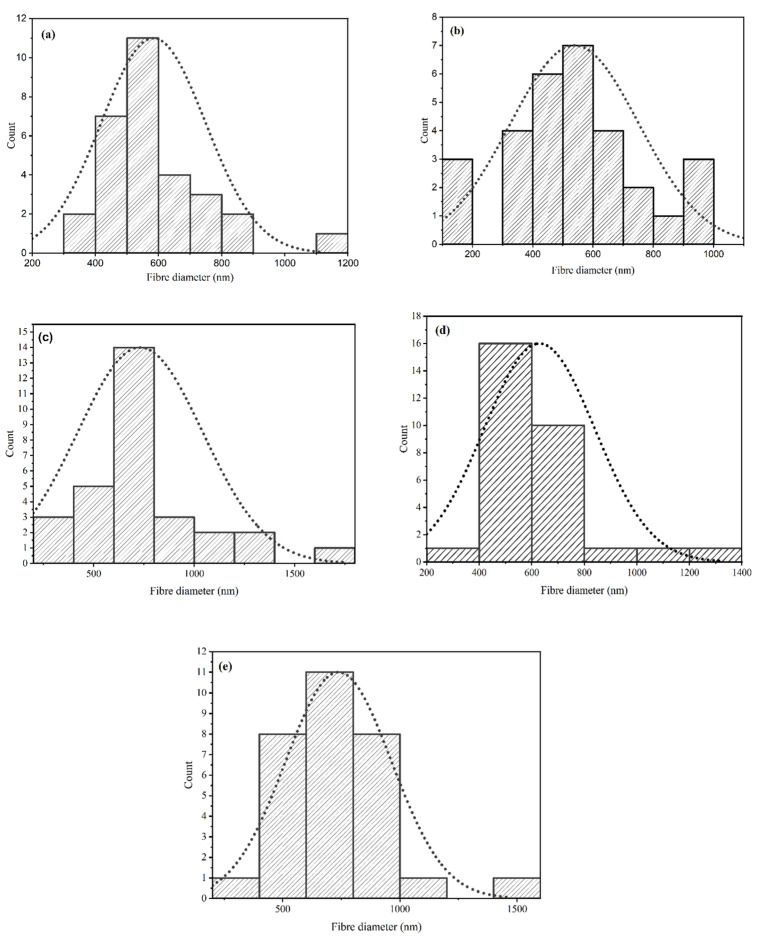
Histograms with distribution curves (dashed line) of fibre diameters (nm) in samples (a–e), *n* = 30. (**a**) CS/PVP only with addition of (**b**) 0.1% *w*/*v* GO, (**c**) 0.2% *w*/*v* GO, (**d**) 0.5% *w*/*v* GO, and (**e**) 0.7% *w*/*v* GO.

Considering Figure 2, skewness calculations showed that all samples were positively skewed (>0) ((a): 1.313, (b): 0.412, (c): 1.28, (d) 1.737, and (e) 1.369), which implies that the right side of the distribution is longer than the left. This is attributed to outliers that lay on the right side of the distribution.

**Figure 3:**
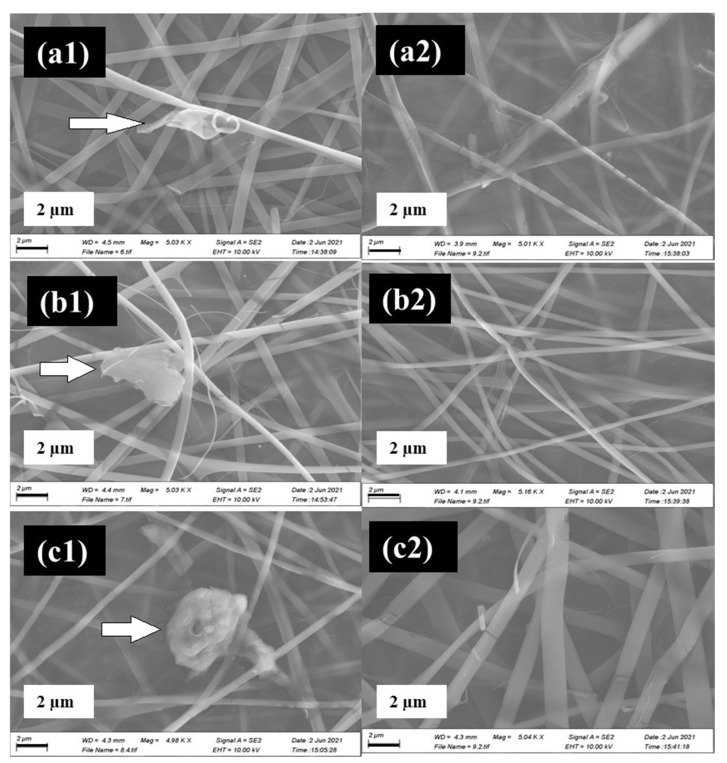
SEM images of samples (a1) to (c1) (containing 0.2% GO-) and samples from (a2) to (c2) without GO under 5 K magnification. All samples contain 4% CS, 6% PVP with various 5-Fu concentrations of 1 mg/mL (**a1**,**a2**), 5 mg/mL (**b1**,**b2**), and 10 mg/mL (**c1**,**c2**). Arrows indicate GO.

Figure 3 provides a side by side comparison of SEM micrographs of samples containing 1 mg/mL, 5 mg/mL, and 10 mg/mL 5-Fu, with and without GO in the polymer blend. GO can be seen in samples (a1), (b1), and (c1) through a wrapped coating and some deposition in between the fibrous matrix. Fibres in the GO-containing samples exhibit homogenous smooth morphologies.

**Figure 4:**
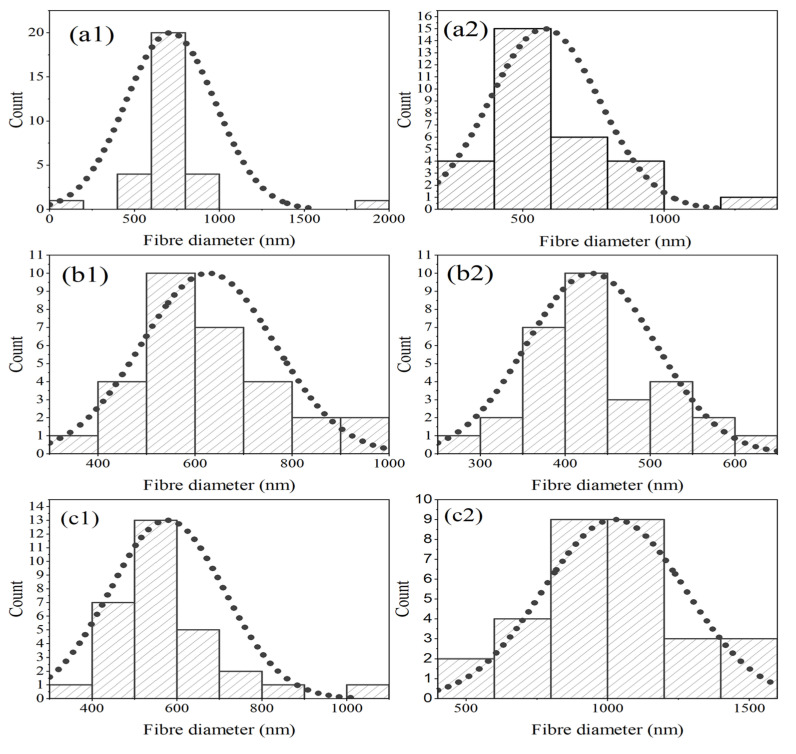
Histograms with normal distribution curves (dashed line) of fibre diameters (nm) in samples (a1) to (c1) and (a2) to (c2), *n* = 30. All samples contain 4% CS, 6% PVP with various 5-Fu concentrations of 1 mg/mL (**a1**,**a2**), 5 mg/mL (**b1**,**b2**), and 10 mg/mL (**c1**,**c2**).

Regarding the GO containing samples (a1) and (a2), both exhibit uniform fibrous morphologies in a random inter-webbed structure. Dissimilarly, sample (a2) possesses roughness on the fibre surfaces. In a direct comparison of samples with GO to those without GO, (a1) and (b1) have higher average fibre diameters than their counterparts ((a2 and (b2)), which has previously been linked to the presence of GO in the matrix [33]. However, it is observed that once 5-Fu drug loading reaches 10 mg/mL (samples (c1) and (c2)), the opposite is true. (c2), which does not possess GO, has a mean average fibre diameter (1024.27 nm) almost double that of (c1) (577.2 nm). It has been suggested that the drug, which is polar in nature, increases the viscosity of the blend, pre-electrospinning. The presence of GO in the matrix may have improved some of the drug adsorption ability and thus stabilized the solution parameters [36]. See Table S3 for mean fibre diameters. Considering the distribution graphs in Figure 4, it is noted that samples (a1) to (c1) (skewness = 2.35, 0.72, 1.91 respectively) and (a2), (b2) (skewness = 1.59, 0.44) were all positively skewed, while sample (c2) was negatively skewed (skewness = −0.128).

### 3.2. XPS Analysis

**Figure 5:**
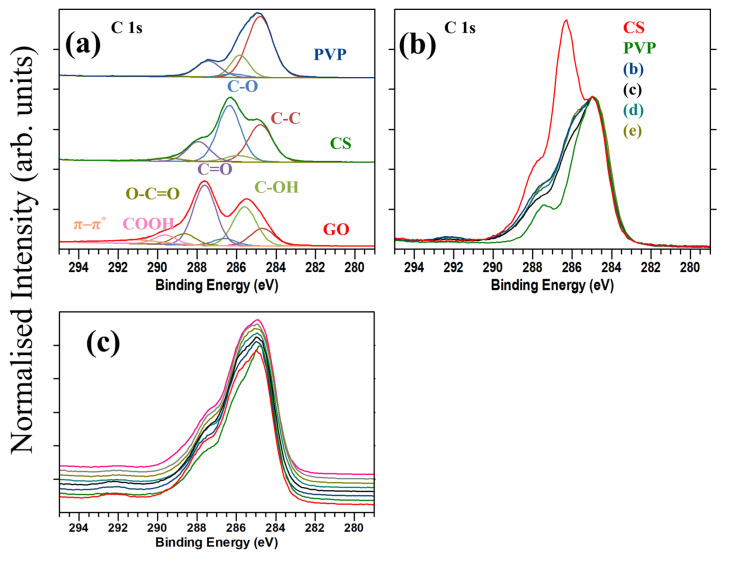
The figure demonstrates (**a**) the deconvoluted C1s peaks of pure GO, pure PVP, and pure CS and (**b**) C1s peaks of samples (b) to (e). Below, (**c**) C1s peaks of samples (a) to (e) are indicated, overlayed with (b1), (b2), (c1) and (c2).

C1s XPS measurements were initially taken for pure GO, pure PVP, and pure CS (see Figure 5). Characteristic bonds were found for pure GO, pure PVP, and pure CS.

It is possible to distinguish carbon signals from CS and PVP in the polymer matrix (see Figure 5). The C-O bonding characteristic of CS is present at approximately 286.3 eV, whereas a more prominent C-OH peak is observed in the case of PVP, as observed in Figure 5a [37]. The overlay of the C1s spectra from the four GO containing samples is shown in Figure 5b, and while there are some subtle differences, in general, the profiles look very similar, the line shape consistent with a combination of CS and PVP in the expected ratio. The concentration of GO in the samples is too low to confidently determine its presence in the samples by XPS due to the overlap of the GO related peaks with those of CS and PVP, as evident in Figure 5a. This is also the case for the samples with the addition of the varying concentrations of 5-Fu, as shown in Figure 5c. The C1s XPS profiles from all samples are still dominated by signals from the CS and PVP. However, this does at least indicate that there is good consistency between the relative amounts of the polymer matrix within all the samples.

Due to the complex nature of the peak fit and the low concentration of GO in the samples, it is not possible to distinguish the presence of the GO signal from that of the CS and the PVP (see Figure 5). Gengenbach et al. [38] highlighted potential mistakes when measuring graphitic and non-graphitic carbon. It is essential not to over-fit the peaks or use a wide range of peak widths, without justification. Graphitic and non-graphitic carbon are generally separated by approximately 0.4 eV to 0.6 eV, resulting in strong peak overlap, especially in our samples where GO is added in such a low concentration [39].

From the compositional analysis of the samples, as shown in Figure S1, the Nitrogen and Oxygen atomic concentration are broadly similar for all the GO samples. Comparing the O concentration from the GO-containing samples (~20%) to that of the individual polymer materials allows us to roughly approximate the relative concentration of each individual polymer in the matrix (CS: ~30%, PVP: ~11%), which suggests a roughly 1:1 ratio over the measured thickness by XPS. Considering XPS is inherently surface sensitive, with 90% of the signal coming from the first 3 nm from the surface, and that the expected ratio based on the source materials is 2 (CS):3 (PVP), this suggests that the surface of the electrospun material could be CS rich. A similar trend is evident when comparing the N at.%.

The F at.% concentration (which is not present in the raw GO, and at trace levels in CS and PVP on their own) increases when the GO is mixed with CS and PVP, suggesting this is evidence of residual solvent remaining in the samples from the fabrication of the CS:PVP matrix [40]. The 0.2% GO sample has the lowest F at.%, which is likely due to variation during the CS:PVP electrospinning.

#### 3.2.1. FTIR Analysis

**Figure 6:**
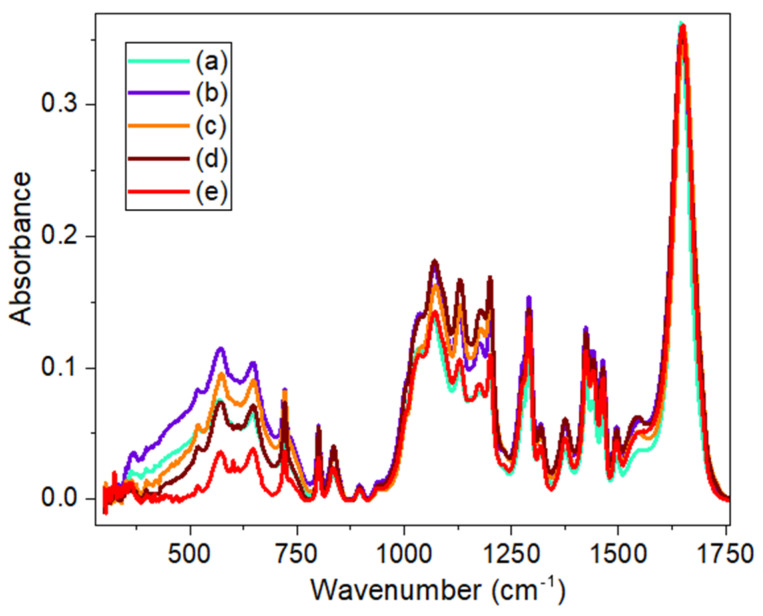
Comparison of FTIR-UATR spectra normalised to C=O peak at ~1650 cm^−1^ for samples with various GO content: (a)—0%GO, (b)—0.1%GO, (c)—0.2%GO, (d)—0.5%GO and (e)—0.7%GO.

Figure 6 shows the spectra of the samples listed in Table S2 (namely samples from (a) to (e)) in the region of 300–1800 cm^−1^ after normalization of all spectra to the intensity of the carbonyl (C=O) band at 1650 cm^−1^. These samples have different concentrations of the graphene oxide (GO) component in the *x*%GO/40%CS/60%PVP composite, where *x* = 0, 0.1, 0.2, 0.5, 0.7%. As can be seen from Figure 6 and Figure S2 (with detailed graphs in the low- and high-frequency ranges), as the content of GO increases, these spectra do not show any unique bands that could be attributed to GO. In the high-frequency part of the IR spectra, no features associated with the content of GO were also revealed. As was shown in our previous work [14], the effect of the presence of GO is more pronounced in the Raman spectra or through the deconvolution of bands in the high-frequency region, and this is discussed in the section on Raman studies of all samples.

The vibrational bands in the IR spectra of these composite samples mainly consist of the contribution of the PVP and CS vibrational bands, which are described in detail in a previous study [14] and elsewhere in the literature [41,42,43]. The main and most intense bands observed in these spectra refer to the absorption band of amide I (stretching vibrations C=O) at about 1650 cm^−1^ (which overlaps for the PVP and CS chemical groups), the absorption bands of the -CONH- and -C-N groups, present at 1068 and 1029 cm^−1^, respectively, while -OH- and -NH- deformations appear in the region of 3200–3500 cm^−1^, and deformations of С-Н groups in the region of 2850–2970 cm^−1^. Other bands appeared in the FTIR spectra related to the angular deformation of the C-H group (bonded to the carbonyl carbon) at 1421 cm^−1^, to the amide III (C=O-NH_2_ axial deformation) at 1317 cm^−1^, to the C-N deformation from the aromatic ring of PVP at 1284 cm^−1^ and C-O angular deformation from CS at 1060 cm^−1^ (see [14,41,42,43] and references therein).

We note that the position of the C=O band differs significantly in the two FTIR measurement methods used in this work by almost 10 cm^−1^ (from ~1650 to ~1660 cm^−1^). This may be due to the sliding of the beam over the sample surface in the case of GATR and multiple reflections at a smaller angle of incidence and, consequently, a greater penetration depth in the case of the UATR tip. In addition, it seems that with the GATR attachments, the surface area from which the IR spectrum is taken is significantly larger than with the UATR tip (in this case, only a small part of the sample located on the tip of the pressure applicator is in contact with the diamond ATR element). At the same time, this difference in the positions of the peaks is practically insignificant in the low-frequency and high-frequency parts of the C=O band. Such a shift of the carbonyl band can be associated with the oxidation of the surface of the samples after long-term storage under atmospheric conditions.

Our attempt to analyze the high-frequency range of the spectra after deconvolution of this spectrum in the region of 2800–3700 cm^−1^, shown in detail in Figure S2b, into several bands also did not offer any reliable results on the GO content.

#### 3.2.2. 5-Fu Content

For a possible assessment of 5-Fu (based on fluorinated pyrimidine) content in the composite from infrared spectra, two sets of samples were analyzed based on composites 0.2%GO/4%CS/6%PVP/*y*5-Fu and 4%CS/6%PVP/y5-Fu with *y* = 1, 5, 10 mg/mL. The investigated samples are listed in Table S1 as (a1), (b1), (c1) for the first set and (a2), (b2), and (c2) for the second set of samples. We note that the base structure with *y* = 0 of 5-Fu for the first set of samples (Table S1) is sample © listed in Table S2.

**Figure 7:**
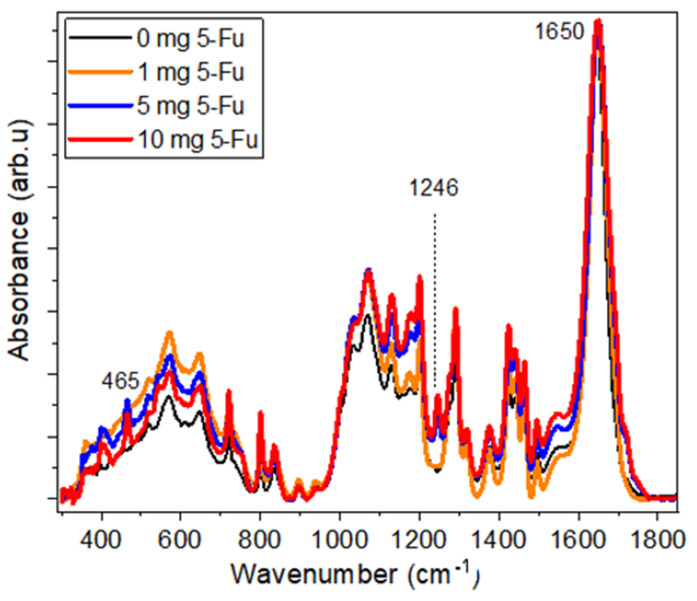
Comparison of FTIR-UATR spectra of samples (c)—0 mg/mL 5-Fu, (a1)—1 mg/mL 5-Fu, (b1)—5 mg/mL 5-Fu, and (c1)—10 mg/mL 5-Fu.

The IR spectra for samples (c), (a1), (b1), and (c1) are shown in detail in Figure 7 in the region of 270–1760 cm^−1^ and in Figure S3 in the low- and high-frequency ranges. These figures show several distinctive features with increasing 5-Fu content: (i) shoulders on the high-frequency side of the C=O band (at ~1650 cm^−1^) at ~1690 and ~1720 cm^−1^, (ii) a very distinct band at 1246 cm^−1^, as well as (iii) several bands with growing intensity upon the increase of 5-Fu content in the region from 300 to 600 cm^−1^.

In accordance with previous theoretical and experimental data [44,45,46,47], these bands are assigned to stretching vibrations of (C2=O) and (C4=O) at ~ 1720 cm^−1^, to C-F and the pyrimidine ring (hereinafter simply as a ring) stretching vibration at 1224–1250 cm^−1^, (OCCF) and ring in-plane bending (at ~541–551 cm^−1^), (C-F), (C=O) and ring in-plane bending at around 467 cm^−1^ (see also Table S4 with 5-Fu vibrational bands and their assignment for IR and Raman spectra from references [44,45]). We note that most of the features listed above associated with the content of 5-Fu were observed in both types of IR spectra measured with GATR and UATR attachments on different FTIR instruments, except for the very-low-frequency range (below 650 cm^–1^) and noisy slopes of the C=O band due to the presence of moisture in the sample compartment in the case of using the GATR attachment.

**Figure 8:**
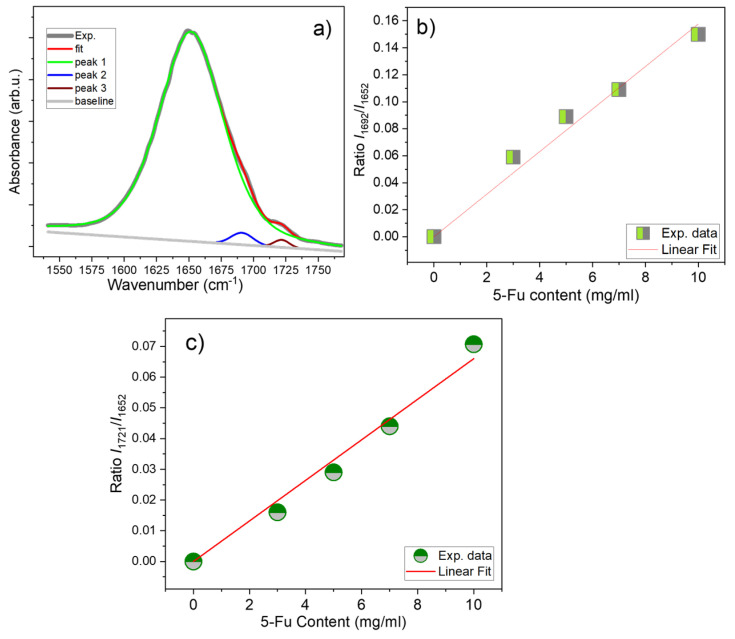
(**a**) Fitting of C=O band with 3 peaks (1652, 1692 and 1721 cm^−1^) for sample (c2) (10 mg of 5-Fu in 4%CS/6%PVP), the dependences of ratio of intensities (**b**) *I*_1692_/*I*_1652_ and (**c**) *I*_1721_/*I*_1652_ vs. 5-Fu content obtained after fitting of FTIR spectra for samples (b1), (c1) (from Table S1), and 2 samples with intermediate 5-Fu content, 3 and 7 mg/mL.

To analyze a slope from the high-frequency side of C=O band, the IR spectra, registered with UATR, were truncated in the region of 1580–1760 cm^−1^, then baselined, normalised to the intensity of the C=O band, and fitted with three bands at 1652, 1692, and 1721 cm^−1^. The result of this fitting itself, and then in the form of plots of the ratio of the intensities of the *I*_1692_/*I*_1652_ and *I*_1721_/*I*_1652_ peaks on the content of 5-Fu (*x*), was plotted and is shown in Figure 8a–c.

The results obtained for two additional samples with intermediate 5-Fu content (with 3 and 7 mg of 5-Fu, respectively) are also included in Figure 8b,c. This was performed in order to increase a number of data points, because a very low intensity of bands at 1692 and 1721 cm^−1^ does not allow for the use of data for sample (b) with 1 mg/mL of 5-Fu. As can be seen from these figures, both graphs show a linear dependence on *x*. We note that the ratios *I*_1692_/*I*_1652_ and *I*_1721_/*I*_1652_ were obtained with the assumption that a small difference in the percentage of GO present in these composites does not significantly affect the intensity of the C=O band (which is mainly resulted from PVP/CS matrix).

**Figure 9:**
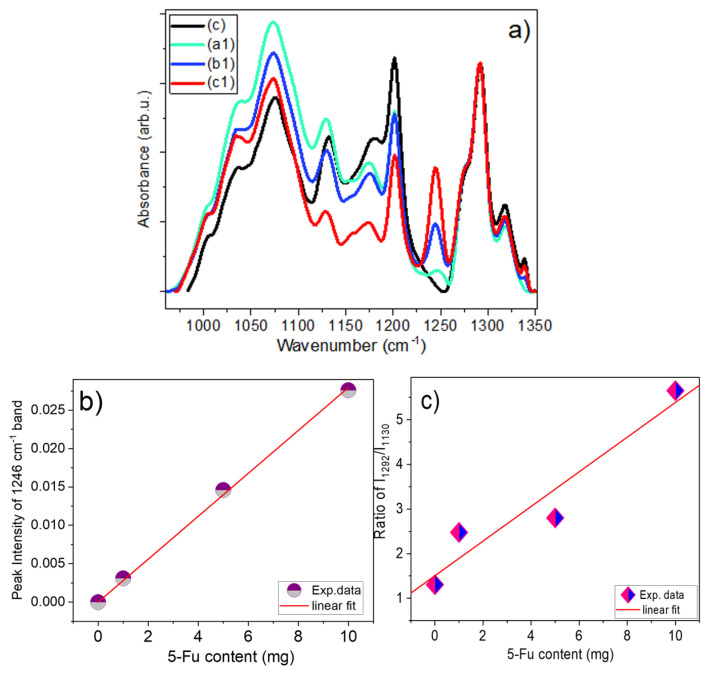
(**a**) FTIR-GATR spectra of samples (c), (a1), (b1), and (c1), truncated in the region of 964–1350 cm^−1^ and then normalized to the peak at 1292 cm^−1^. The dependence of (**b**) peak position of 1246 cm^−1^ band and (**c**) the ratio of peak intensities *I*_1292_/*I*_1130_ vs. 5-Fu content.

We note that vibrational bands near 1246 cm^–1^ and 1721 cm^–1^ were also observed in the FTIR spectra of various composites (including fibrous ones) with 5-Fu used for some biomedical applications in [48,49,50,51,52,53,54]. The 1246 cm^−1^ band in this present work was analyzed using the data obtained from both spectrometers. However, as an example, the results of the analysis of IR spectra recorded using the GATR attachment are used below. The FTIR-GATR spectra for samples (c), (a1), (b1), and (c1) were truncated in the range of 964–1347 cm^−1^, baselined, and then normalized to the intensity of the band at 1292 cm^−1^ as shown in Figure 9a. The latter was performed because, after normalizing the spectra by the intensity of the peak at 1650 cm^−1^, this was the only peak that had a very similar intensity for all these samples on the one hand and was located very close to the discussed peak at 1246 cm^−1^ on the other. This part of the spectra demonstrates several features, namely (i) a very pronounced appearance of a peak at 1246 cm^−1^ at a higher content of 5-Fu; (ii) a sharp decrease in the relative intensity of the 1130 cm^−1^ band with respect to the 1073 cm^−1^ band; as well as the 1292 cm^−1^ band.

For a correct assessment of the intensity of the band at 1246 cm^−1^, the spectra shown in Figure 9a were fitted in the manner shown in Figure S4, and the results obtained after this fitting are shown in Figure 9b as a linear dependence of 1246 cm^−1^ peak intensity vs. 5-Fu content, *x*, and in Figure 9c as a dependence of the *I*_1292_/*I*_1130_ intensity ratio vs. *x*. Both graphs demonstrate the linear dependence of these values and can serve as a basis for their use for calibrating the content of 5-Fu in fibre composites. A similar result was obtained for the spectra recorded with the UATR attachment on the Perkin Elmer spectrometer, as shown in Figure S5a,b.

**Figure 10:**
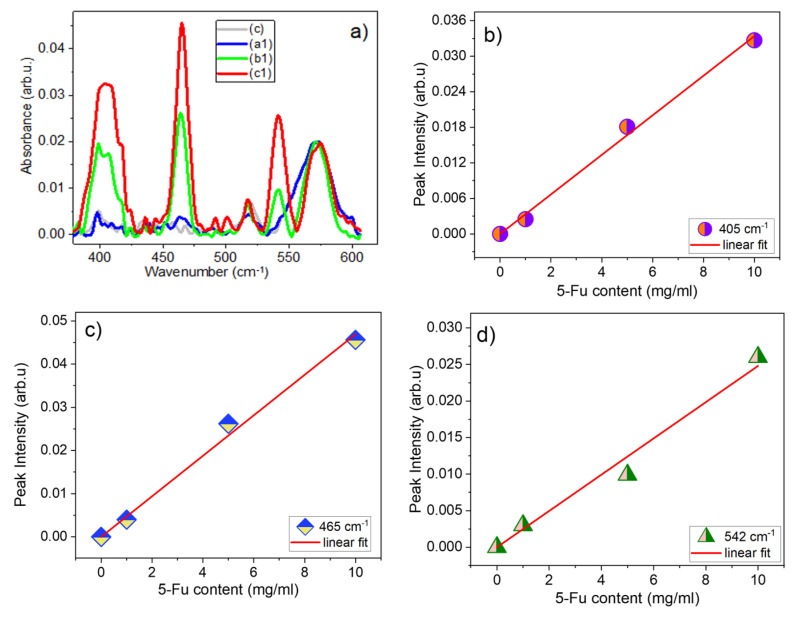
(**a**) Baselined region of samples (c), (a1), (b1), and (c1), normalized to the intensity of 572 cm^−1^ peak, for analysis of low-frequency vibrations. The dependence of peak position of low-frequency bands vs. 5-Fu content estimated from FTIR spectra, shown in figure (**a**), for (**b**) 405 cm^−1^, (**c**) 465 cm^−1^, and (**d**) 542 cm^−1^ bands.

In addition to the above, quite interesting results have been demonstrated for the low-frequency bands in the 370–650 cm^−1^ region, which were recorded using only the FTIR-UATR method. In particular, as can be seen from Figure 10a, several bands in this region located at 542, 465, and 405 cm^−1^ also show an increase in intensity with increasing 5-Fu content. These peak positions are in accordance with the corresponding bands observed in the literature and the calculated vibrational frequencies (see Tables S4 and S5 in Supporting Information) for pure 5-Fu and its various compounds [44,45,46,48]. The dependences of the peak intensities of these bands after fitting of this region of the spectrum are shown in Figure 10b–d, and all of them show a linear dependence on the content of 5-Fu. Similar results are also shown for samples (a), (a2), (b2), and (c2), which are shown in Figure S6a–d. Furthermore, a comparison of the dependences of these three peaks on 5-Fu content for samples with and without GO is shown in Figures S7a–c, which demonstrates that the slopes for all three peaks for samples with 0.2% GO are on average 1.6 times higher than for samples without GO. This can be used in future work to evaluate the number of parameters required for biomedical applications of different drug delivery vehicles.

**Figure 11:**
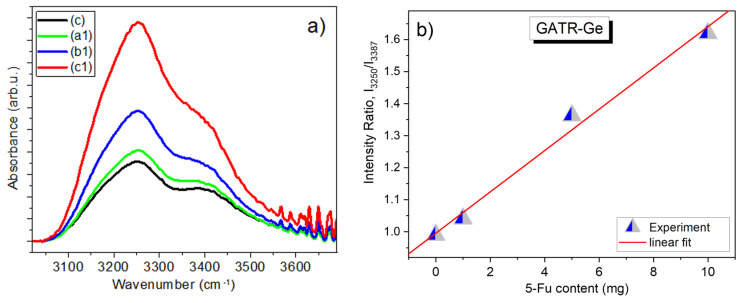
(**a**) High-frequency part of FTIR-GATR spectra of samples (c), (a1), (b1), and (c1). (**b**) The dependence of the ratio of peak intensities *I*_3250_/*I*_3387_ vs. 5-Fu content.

Finally, the high-frequency range of the spectra recorded with the GATR attachment was also analyzed after fitting all the spectra shown in Figure 11a with three bands around 3160, 3250, and 3387 cm^−1^ (as shown in Figure S8). The ratio of the peak intensities of the bands at 3250 and 3387 cm^−1^ is plotted as a function of the 5-Fu content in the graph shown in Figure 11b and shows a linear relationship with 5-Fu content.

In conclusion, it should be noted that in a detailed analysis of the IR spectra recorded using two FTIR spectrometers, several options are available for determining the concentration of 5-Fu in composite fibre systems 4%CS/6%PVP/*y*5-Fu and 0.2%GO/4%CS/6%PVP/*y*5-Fu (when *y* = 0, 1, 5, 10). These are the following options: (i) use the peak intensity of the 1246 cm^−1^ band, (ii) use the intensity of one of the low-frequency bands 405, 465, and 543 cm^−1^ (if the FTIR spectrometer allows measuring this range of spectra); (iii) use intensities of the 1692 and 1721 cm^−1^ bands on the high-frequency side of the C=O band; and (iv) use the ratio of the intensities of the high-frequency peaks obtained after deconvolution of the 3000–3800 cm^−1^ range of spectra. It should be noted that when using Options (ii) and (iii), it is necessary to normalize the spectra to the intensity of the C=O band at 1650 cm^−1^ or, even better, to the intensity of the band at 1292 cm^−1^, which belongs to the fibrous composite. Another important point to be emphasized is that the intensity of the 1246 cm^−1^ band should be estimated after fitting all the bands together in the 964–1347 cm^−1^ range, preliminarily baselined on these two boundary points.

### 3.3. Raman Analysis of GO Content

Currently, a significant amount of work is being carried out on the study of 5-Fu and its derivatives dissolved in biological fluids (such as saliva), as well as in composite materials used for biomedical applications (see, for example, [43,48,49,51,53,55,56]). For these studies, conventional Raman spectroscopy was used, as well as various types of enhanced Raman spectroscopy. As mentioned above, in the present work, we used micro-Raman spectroscopy to study fibrous composites with inserted GO clusters and/or 5-Fu drug. The Raman spectra were collected from different points, namely by focusing the laser beam on the fibres, as shown in Figure S9a, or, in some cases, onto the visible GO cluster (see Figure S9b). Because of this, the Raman spectra differed in the region of the D and G bands of GO. In addition, in many cases, a rather significant fluorescence background was observed, which made it difficult to obtain spectra in a wide range (namely from 200 to 3600 cm^−1^). Therefore, some spectra were registered separately in the low- and high-frequency ranges with a long accumulation time (up to 700–1000 s). As a result, the Raman spectra in many cases were quite noisy.

**Figure 12:**
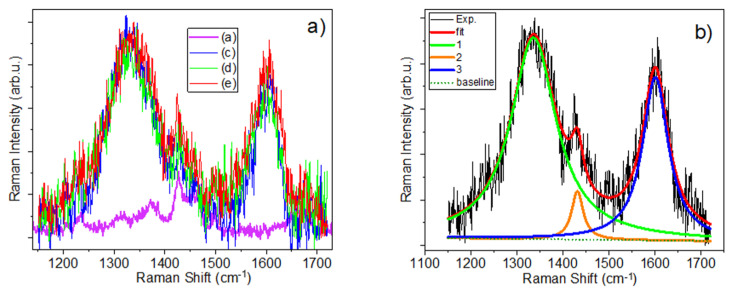
(**a**) Raman spectra of (a), (c), (d), and (e) samples shown in the range of D and G bands of GO, (**b**) example of fitting of Raman spectrum of sample (e) with three bands.

Regarding the Raman spectra of samples (a)–(e) with different contents of GO, it can be seen from Table S2 that the fibre diameter for sample (b) with 0.1% GO is the smallest, which leads to difficulties in Raman measurements. We found that better results during spectra registration were obtained when the laser beam was focused on individual fibres (see Figure S9a,b). Thus, smaller sample diameter creates some problems with spectra registration. As mentioned above, due to the strong fluorescence background, Raman measurements were carried out separately for the low and high frequencies. The obtained Raman spectra are shown in Figure 12a,b. It can be seen from this figure that the Raman spectra of the GO/Cs/PVP composite show a strong presence of GO in the region of the D and G bands at 1335 and 1605 cm^–1^, in contrast to the IR spectra of these samples. At the same time, in Figure S10, we can clearly see the appearance of some bands that belong to the CS/PVP fibre matrix. In particular, the band around 1425 cm^−1^ is the most pronounced, some other bands also appear at 1378, 1456, and 1495 cm^−1^ and on the low-frequency slope of the D-bands around 1238 cm^−1^. It is also noticeable that the intensity of the 1425 cm^−1^ band decreases with increasing GO content. The deconvolution of the bands in the region of 1250–1750 cm^−1^ was carried out in two ways: (i) using six bands for fitting and (ii) in a simpler way, using only three bands for fitting at 1335, 1425, and 1605 cm^−1^ (see Figure 12b), because the 1425 cm^−1^ band shows the highest intensity.

**Figure 13:**
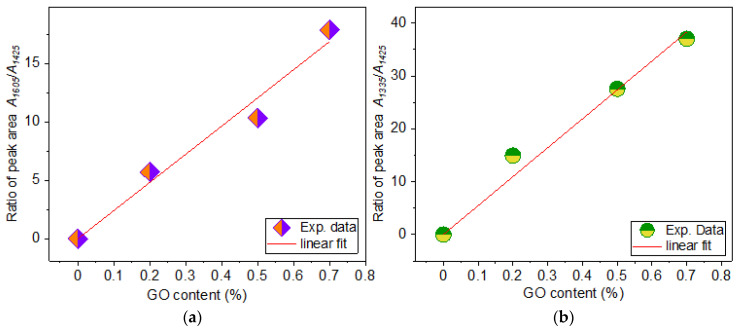
The ratio of integrated intensities (**a**) *A*_1425_/*A*_1605_ and (**b**) *A*_1425_/*A*_1335_ vs. GO content for samples (a)–(e) after fitting the spectral range 1150–1750 cm^−1^ with three bands.

Fitting of this region with three bands (as well as with six bands) results in dependences of A_1335_/A_1425_ and A_1605_/A_1425_ vs. *x*, as shown in Figure 13 (and in Figure S11 after fitting with six bands). As one can see, these relationships demonstrate a linear dependence with an increase in the content of GO. This is in addition to what has already been demonstrated in the literature for estimating GO content in other matrices using a linear dependence of *I_D_*/*I_G_* on GO content [57]. In the case of the samples studied in this work, this approach did not work very well. This could be due to the influence of other vibrational bands from the CS/PVP matrix appearing on the slopes of D and G bands, which affects their fitting.

Regarding the use of micro-Raman spectroscopy to evaluate the 5-Fu content in our samples, we found that it is difficult to use this approach to determine the 5-Fu content in GO/PVP/PS or even in the PVP/CS matrix due to the relatively low Raman signal from the entire system and to the difficulties to detect weak Raman bands from 5-Fu on the background higher intensity bands from the host structure and quite noisy Raman spectrum. This contrasts with FTIR measurements where several relatively weak bands could be detected even for the mentioned 5-Fu concentrations. Deconvolution of the high-frequency Raman bands also did not provide additional information on the content of 5-Fu in both matrices.

### 3.4. Cytotoxicity Analysis

#### 3.4.1. Samples (a–e)

**Figure 14:**
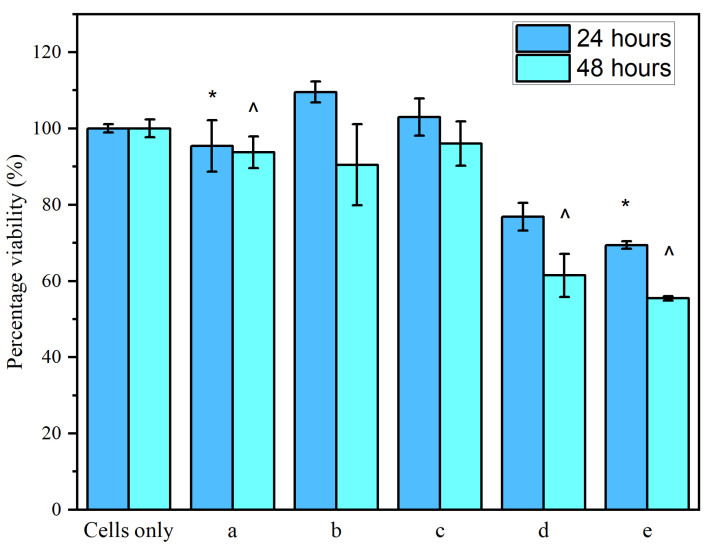
The cell viability of CaCo-2 cells when exposed to samples (a) (CS/PVP), (b) (0.1 *w*/*v*% GO in CS/PVP), (c) (0.2 *w*/*v*% GO in CS/PVP), (d) (0.5 *w*/*v*% GO in CS/PVP), and (e) (0.7 *w*/*v*% GO in CS/PVP) over 24 and 48 h. * and ^ denote statistical significance between CS/PVP control and experiments groups containing GO two-sample *t*-test, *p* < 0.05, *n* = 3.

Several significant observations were made in the cytotoxicity results (see Figure 14). Over 24 h of exposure, sample (a) (CS/PVP) (95.41% viability) was statistically non-different to cells only (*p* = 0.58) (100% viability), signifying a biocompatible material in the CS/PVP construct. While sample (d) (0.5 *w*/*v*% GO in CS/PVP) showed insignificant differences to sample (a) (CS/PVP) (*p* = 0.068), its viability (76.84%) was just under the 80% biocompatible threshold [58]. Sample (e) (0.7 *w*/*v*% GO in CS/PVP) showed a statistically significant cell kill (69.4% viability) compared to sample (a) (*p* = 0.027). This means that the GO concentration of 0.7 *w*/*v*% GO constructs was shown as toxic after 24 h exposure.

In addition, 48 h exposure furthered our understanding of potential cytotoxicity within the GO range. Again, sample a was shown to be non-toxic after 48 h (93.77% viability) compared to cells only (*p* = 0.288). Non-toxicity of samples (b) (90.48% viability) (0.1% *w*/*v* GO) and (c) (96% viability) (0.2% *w*/*v*) compared to sample (a) (CS/PVP) was shown (*p* = 0.807 and 0.153, respectively). The viability of samples (d) (61.47%) and (e) (55.48%) versus sample (a) was shown to be statistically significant (*p* = 0.00453 and 0.00126, respectively), thus indicating 48 h toxicity once a concentration of 0.5% *w*/*v* GO and above was reached. This allowed us to narrow our GO concentration margin to between 0.1% *w*/*v* and <0.5% *w*/*v*.

At low doses, we can see that GO is inherently non-toxic to CaCo-2 cells in these CS/PVP constructs. Another study analyzed 3 µg/mL of GO (which falls just above our 0.2% sample which is roughly a 2 µg/mL dose) dispersed in media and applied to CaCo-2 cells. After 24 and 78 h exposure, their sample showed non-toxicity, comparable to our sample [59]. It is hypothesized that the surface functionality of GO shields hydrophobic domains [59]. Due to the oxygen-rich functional groups on the surface of GO, the material may pose high potential as a nanocarrier for designing drug delivery systems.

A recent study examined the exposure of GO to a co-culture of CaCo-2/HT29 cells which mimic the intestinal epithelial characteristics [60]. Samples within and above our own GO range (our 0.7% *w*/*v* equals approximately 7 µg/mL GO, assuming full homogeneity) have shown a constant, high cell viability in their 0 to 100 µg/mL range [60]. This is only true for our 0.1%, 0.2%, and 0.5% GO samples ((b), (c), and (d)), which may suggest that GO loaded into electrospun constructs has different toxicity mechanisms to regular, dispersed GO in the medium or that the HT29 cells in the co-culture have a better affinity for GO. This is why our cytotoxicity analysis of a GO range was important before assessing drug loading/release for the construction of an anti-cancer delivery vehicle.

#### 3.4.2. 5-Fu-Containing Samples (a1–c1, a2–c2)

**Figure 15:**
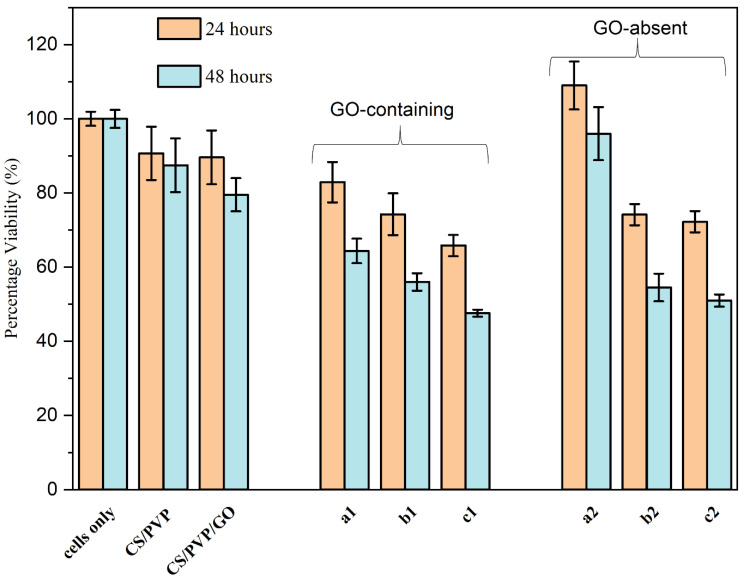
The viability of CaCo-2 cells exposed to electrospun constructs over 24 and 48 h. All samples contain 4% CS, 6% PVP. Variables are as follows: (a1) 0.2% GO—1 mg/mL 5-Fu; (a2) 1 mg/mL 5-Fu; (b1) 0.2% GO—5 mg/mL 5-Fu; (b2) 5 mg/mL 5-Fu; (c1) 0.2% GO—10 mg/mL 5-Fu; (c2) 4% CS, 6% PVP—10 mg/mL 5-Fu.

Further, 0.2% *w*/*v* GO was chosen to be loaded into CS/PVP (6:4) scaffolds from our previous findings, with only the voltage and direct current, both being adjusted to 15 kV. This was performed to allow for spinning of samples with 5-Fu due to added viscosity of the mixture in order to construct a drug delivery vehicle consisting of CS/PVP/GO/5-Fu and compare it to CS/PVP/5F-u (no GO). Figure 15 details the results of these findings. t-tests assuming unequal variances were applied to the data to check significant differences in cell viability between samples. Firstly, the control containing only CS/PVP showed no significant differences to cells only at both 24 and 48 h. This was similar to the CS/PVP/GO control which showed no differences at 24 h to cells only. At 48 h, there was a slight significant difference; however, the viability was shown at 79.51%, which is approaching the 80% biocompatible threshold.

Regarding Table 1, we can see that the GO-containing samples ((a1), (b1), and (c1)) all present significant kill over 48 h against the three controls.

Samples (a1), (b1), and (c1) were loaded with 1 mg/mL, 5 mg/mL, and 10 mg/mL 5-Fu, respectively (with CS/PVP/0.2%GO). A regressive cell kill was observed at both 24 h and 48 h for the three samples (82.89%, 74.25%, 65.81%—24 h, 65.35%, 55.97%, and 47.56%—48 h, respectively). Significant differences were found between sample (a1) (1 mg/mL 5-Fu) and sample (c1) (10 mg/mL 5-Fu), *p* = 0.047. Over 48 h, significant differences were found between samples (a1) and (c1) (*p* = 0.0072) and samples (b1) and (c1) (0.032).

Samples (a2), (b2), and (c2) were similarly loaded with 1 mg/mL, 5 mg/mL, and 10 mg/mL 5-Fu as above, this time with no GO present in the CS/PVP electrospun construct. We can see that sample (a2), 1 mg/mL 5-Fu, was not effective at killing cells over 24 and 48 h (109% and 96% viability, respectively). A more prominent kill is then seen with sample (b2) (74.13%—24 h, 54.48—48 h viability); however, this appears to almost plateau when moving from sample (b2) to (c2), over both 24 and 48 h (72.23% and 50.93%). Within this test group, significant differences were observed between sample (a2) and both (b2) (*p* = 0.0097) and (c2) (*p* = 0.0079) at 24 h and between (a2) and b2 at 48 h (*p* = 0.00395).

Comparing samples with GO to those with no GO, first, we can see that the samples with GO present show a statistically significant linear kill, defined by the regression equation *y* = −0.0672*x* + 63.816 (*p* = 0.029). Samples with no GO do not show a statistically significant linear kill. At low doses of 5-Fu, GO-containing samples are more efficient at killing CaCo-2 cells. This is shown when analyzing (a1) vs. (a2), where the t-test presented *p* values of 0.036 and 0.01 after both time points. Samples (b1) and (b2) have very similar cell kill over both time points (74.25% vs. 74.13% at 24 h and 55.97% vs. 54.48% at 48 h, respectively). Finally, when comparing (c1) to (c2), the GO-loaded (c1) sample had an improved cellular kill of 6.42% after 24 h and 3.37% after 48 h compared to sample (c2), which had no GO present. Free 5-Fu in solution (with no GO) was not studied for its effects on cells, as there would be no comparable delivery mechanism without the CS\PVP.

A similar study, which analyzed the release of 5-Fu, DOX and PTX from CS/PLA triaxial nanofibres, highlighted a cellular kill of around 53% after 2 weeks from their single-layer nanofibres [23]. Our work reached and surpassed this threshold after 48 h, as seen in samples (c1) and (c2), both with cellular viability of 47.56% and 50.93%, respectively. Another study has shown that 1 mg/mL of 5-Fu in CS/PVP electrospun constructs was not efficient at killing A549 cells, while a 5-Fu concentration of 10 mg/mL was efficient [14].

### 3.5. Drug Release Analysis

**Figure 16:**
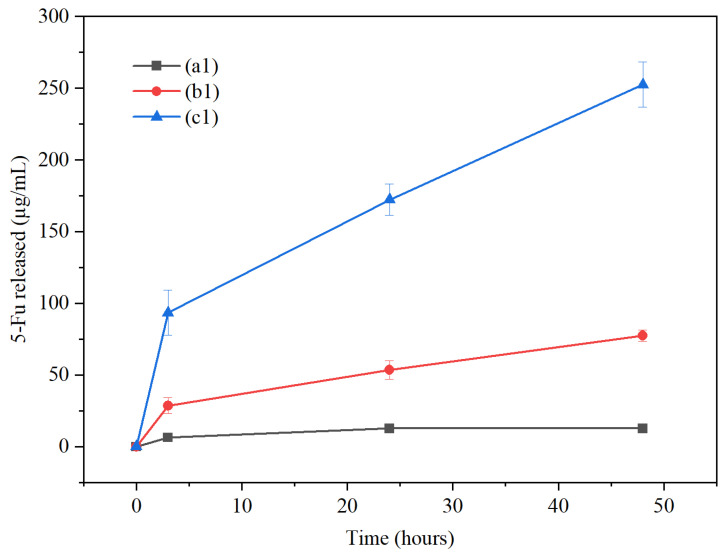
Cumulative 5-Fu released after 3, 24, and 48 h of samples (a1), (b1), and (c1). Samples (a1)—1 mg/mL 5-Fu; (b1)—5 mg/mL 5-Fu; (c1)—10 mg/mL 5-Fu.

Drug release studies were performed on samples containing GO: ((a1)—1 mg/mL 5-Fu), ((b1)—5 mg/mL 5-Fu), and ((c1)—10 mg/mL 5-Fu) to assess their release profiles over 3, 24, and 48 h (see Figure 16). Sample (a1) saw a cumulative release of 5-Fu at 6.38 µg/mL, 12.81 µg/mL, and 12.81 µg/mL (no additional release), sample (b1) released 28.70 µg/mL, 53.64 µg/mL, and 77.41 µg/mL, and sample (c1) released 93.62 µg/mL, 172.36 µg/mL, and 252.65 µg/mL over 3, 24, and 48 h, respectively. A single-factor ANOVA test showed variation within the means of samples (a1), (b1), and (c1) over 3 h (*p* = 0.004803, *n* = 3). Post hoc t-tests (two-sample, assuming unequal variance) highlighted statistical differences between samples (a1) and (c1) (*p* = 0.046). The same testing criteria were applied to both the 24 h and 48 h time points. ANOVA results of each showed variation within the means at each time points (*p* = 0.0038 and 0.0064, respectively). The same post hoc *t*-test showed variation within the means between samples (a1) and (c1) (*p* = 0.036) and (b1) and (c1) (*p* = 0.044) 24 h. At 48 h, variation was not reported between samples (a1) and (b1) and (c1) because the data values at (a1) were all zero (no release since 24 h), thus incorrectly skewing variation.

The 3 h time point measurement for each sample saw an initial “burst release”, which is commonly associated with drug release from nanofibres [61,62], especially 5-Fu, which is hydrophilic [63]. This may also apply to the drug being near the surface of the material, where phase separation can also occur [64]. A slower, more “sustained” release profile was seen for sample (b2), which follows a Fickian diffusion mechanism [61]. Sample (a1), which had the lowest 5-Fu concentration (1 mg/mL), tailored off after 24 h, meaning all of the drug was fully released by then. The high “burst release” of sample (c1) relates to the drug moving through diffusion and dissolution, from a superficial area of the fibres [65].

These results were compared to previous work by Grant et al., 2021, where a similar drug release study was carried out with samples containing 1 mg/mL, 5 mg/mL, and 10 mg/mL of 5-Fu in electrospun composites containing 4% CS/6% PVP (4:6), with no GO present [14] (see Table 2).

**Figure 17:**
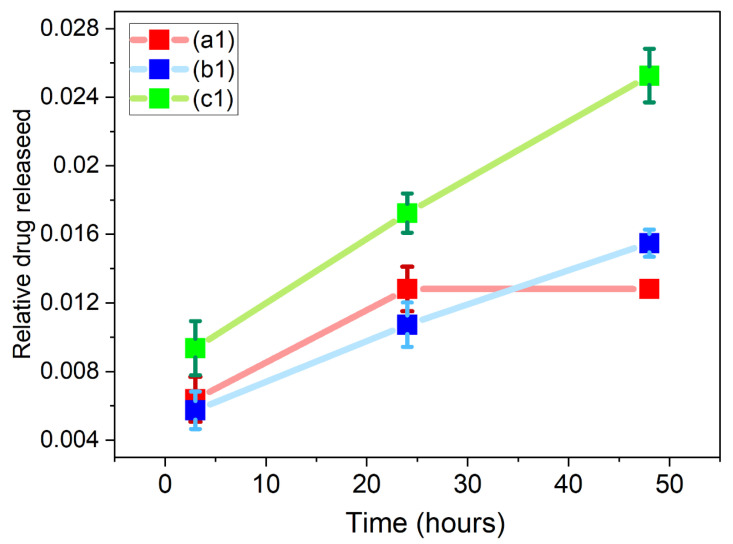
Relative drug release of samples (a1), (b1), and (c1) over 3, 24, and 48 h. Relative drug release is the absolute drug release normalized according to the initial concentration of drug to allow comparison of the drug release rates across the three formulations.

Figure 17 shows the release of 5-Fu relative to drug concentration loaded. As can be seen, the 1 mg/mL and 5 mg/mL concentrations show similar relative release profiles. However, the 10 mg/mL shows a higher relative release profile. A possible reason for this may be more unbound drug particles in the higher concentration.

Samples containing 1 mg/mL 5-Fu have shown a similar release pattern with and without GO [14]. However, as the drug concentration increases to 5 mg/mL, samples with GO present have an improved drug loading/release efficiency, at almost double that of the samples with no GO present. Finally, regarding samples with 10 mg/mL 5-Fu, again, those with GO present better loading/release than those without GO present. Here, we can state that at above 1 mg/mL and up to and including 10 mg/mL 5-Fu concentration, there is overall improved loading/release of the anti-cancer drug in the samples containing GO versus those without. We believe that this result is convincingly supported by the FTIR data presented in Section 3.3 (see Figure 10 and Figures S6 and S7) of this paper on the dependences of the intensities of the three 5-Fu-related peaks on its content in the construct. The slopes for these dependences for the samples with GO are on average 1.6 higher than for those without GO. A more detailed analysis of the FTIR spectra with a parallel development of a method for determining the amount of 5-Fu actually loaded into both sets of samples will allow the use of FTIR data for quantitative analysis of various parameters (such as encapsulation efficiency, drug loading/release, as well as detection limits/quantification of drugs, etc.) for biomedical applications.

**Figure 18:**
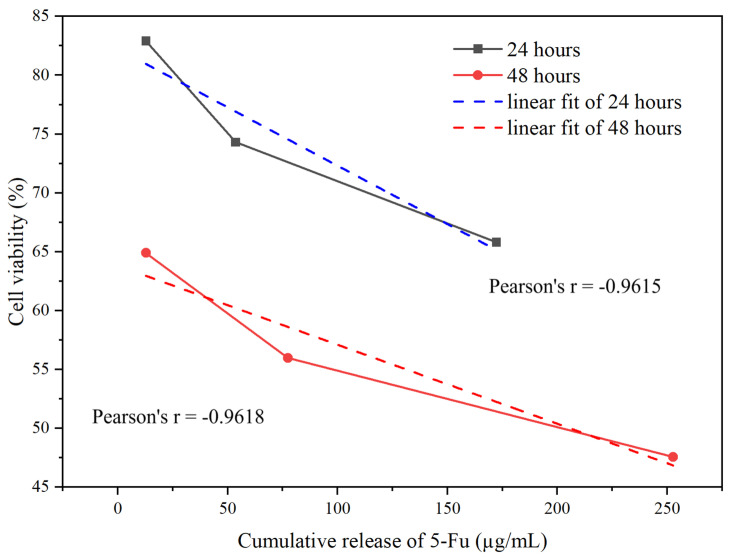
Cell viability (%) versus cumulative release of 5-Fu (µg/mL), over both 24 and 48 h.

There is a negative correlation between cell viability and drug release of samples (a1), (b1), and (c1) over 24 and 48 h. Pearson’s *r* values of −0.9615 and −0.9618 over the two time points signify a strong negative correlation (see Figure 18).

## 4. Conclusions

Upon studying the range of GO concentrations in CS/PVP, the concentration of 0.2% *w*/*v* GO was chosen for inclusion in the drug delivery model. SEM showed bead-free, homogenous fibres within the construct (sample e). This sample also proved non-toxic to CaCo-2 cells over 24 and 48 h exposure. The construction of a drug delivery vehicle whereby 5-Fu was loaded with and without GO in various concentrations showed several interesting findings.

The presence of CS/PVP was revealed through XPS, FTIR, and Raman spectroscopies. A detailed analysis of the IR spectra recorded using two FTIR spectrometers, several options for determining the concentration of 5-Fu in composite fibre systems CS/PVP/5-Fu and GO/CS/PVP/5-Fu, were demonstrated. These options were shown by using (i) the peak intensity of the 1246 cm^−1^ band, (ii) the intensity of the low-frequency bands at 405, 465, and 543 cm^−1^; (iii) intensities of 1692 and 1721 cm^−1^ bands on the high-frequency side of the C=O band; and (iv) the ratio of the intensities of the high-frequency peaks in 3000–3800 cm^−1^ range of spectra. By the analysis of Raman spectra in the region of D and G bands, a linear dependence of ratios of integrated intensities of *A_D_* and *A_G_* to the intensity of the host polymer band at 1425 cm^−1^ vs. GO content was found.

Cytotoxicity analysis showed that at low doses (1 mg/mL 5-Fu), the GO samples had increased kill of CaCo-2 cells (82.89% vs. 109% and 64.35% vs. 96% after 24 and 48 h exposure) with non-GO loaded samples. Drug release showed similar improved results when GO was included in the scaffold. Negative correlation between cytotoxicity vs. drug release was confirmed. This shows that the material could have potential as a drug delivery vehicle for chemotherapeutic purposes (adhered to peri-cancerous tissues during surgeries), targeting colon cancer, negating the effects of 5-Fu administered intravenously.

## Figures and Tables

**Table 1:** List of *p* values in the t-test results matrix, assuming unequal variance, between the samples and the cells only, CS/PVP and CS/PVP/GO controls. Insignificant *p* values are marked with “-”.

24 h	a1 1 mg/mL 5-Fu	b1 5 mg/mL 5-Fu	c1 10 mg/mL 5-Fu	a2 1 mg/mL 5-Fu	b2 5 mg/mL 5-Fu	c2 10 mg/mL 5-Fu
	GO Containing			No GO		
Cells only	0.045	0.011	0.000049	-	0.00011	0.00018
CS/PVP	-	-	0.035	-	0.00027	-
CS/PVP/GO	-	-	0.041	-	-	-
48 h						
Cells only	0.00035	0.00035	0.000018	-	0.00011	0.0000035
CS/PVP	0.041	0.0143	0.0083	-	-	0.012
CS/PVP/GO	0.045	0.006	0.034	-	-	0.003

**Table 2:** Comparison of 5-Fu released from samples (a1), (b1), and (c1) to samples without GO from Ref. [14].

Sample	Concentration of 5-Fu Released (µg/mL)			
5-Fu Concentration (GO-Absent)	3 h	24 h	48 h	Reference
1 mg/ml	10.27	13.98	15.6	[14]
5 mg/ml	14.83	29	41.93	[14]
10 mg/ml	61.12	117.41	169.85	[14]
5-Fu with GO present				
(a1) 1 mg/ml	6.38	12.81	12.90	N/A
(b1) 5 mg/ml	28.70	53.64	77.41	N/A
(c1) 10 mg/ml	93.62	172.36	252.65	N/A

## Data Availability

The original contributions presented in the study are included in the article/Supplementary Materials, further inquiries can be directed to the corresponding author.

## Supplementary Materials

The following supporting information can be downloaded at: https://www.mdpi.com/article/10.3390/ma17215300/s1.
