# Supplementary material for: Enhancement of 5-Fluorouracil Drug Delivery in a Graphene Oxide Containing Electrospun Chitosan/Polyvinylpyrrolidone Construct
Source: Materials (Basel). 2024 Oct 31;17(21):5300. doi: 10.3390/ma17215300 (PMC11548022; doi:10.3390/ma17215300)
Supplement: Supplementary file 1 [file materials-17-05300-s001.zip › materials-3089488-supplementary.pdf]

**Supplementary Information**

**Table S1**: highlights the polymers, GO concentrations and 5-Fu concentration in each sample.

| **Sample Name** | **Polymer** | **GO concentration** | **5-Fu concentration** |
|---|---|---|---|
| **(a1)** | 4% CS/ 6% PVP | 0.2 % w/v | 1 mg/mL |
| **(b1)** | 4% CS/ 6% PVP | 0.2 % w/v | 5 mg/mL |
| **(c1)** | 4% CS/ 6% PVP | 0.2 % w/v | 10 mg/mL |
| **(a2)** | 4% CS/ 6% PVP | - | 1 mg/mL |
| **(b2)** | 4% CS/ 6% PVP | - | 5 mg/mL |
| **(c2)** | 4% CS/ 6% PVP | - | 10 mg/mL |

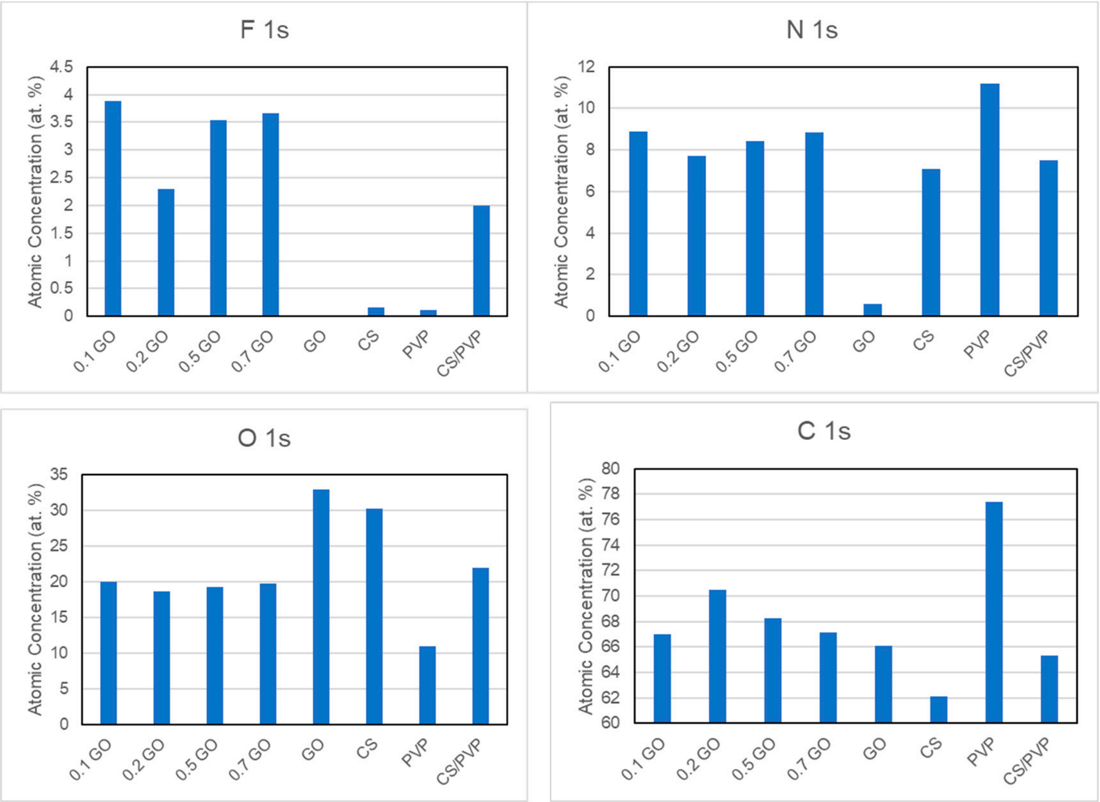


**Figure S1**. Shows the atomic concentration (at %) of Fluorine, Nitrogen, Oxygen and Carbon in samples: pure GO, pure CS, pure PVP (a) to (e). (a) CS/PVP only (b) 0.1 %w/v GO added (c) 0.2 %w/v GO added (d) 0.5 % w/v GO added (e) 0.7 % w/v GO added

**Table S2**: average fibre diameter (nm) of samples **(a)** to **(e)**

| **Sample** | **Average fibre diameter (nm)** |
|---|---|
| **(a)** CS/PVP only | 583 |
| **(b)** 0.1 %w/v GO added | 534 |
| **(c)** 0.2 %w/v GO added | 731 |
| **(d)** 0.5 % w/v GO added | 629 |
| **(e)** 0.7 % w/v GO added | 738 |

**Table S3.** Mean fibre diameter (nm) of samples (a1) to (c1) and (a2) to (c2).

| **Sample** | **Mean fibre diameter (nm)** | **Standard Deviation** |
|---|---|---|
| (a1) 4% CS, 6% PVP, 0.2% GO - 1 mg/mL 5Fu | 712.00 | 259.6900 |
| (a2) 4% CS, 6% PVP - 1 mg/mL 5Fu | 577.43 | 190.2407 |
| (b1) 4% CS, 6% PVP, 0.2% GO - 5 mg/mL 5Fu | 627.20 | 135.6998 |
| (b2) 4% CS, 6% PVP- 5 mg/mL 5Fu | 430.83 | 75.04935 |
| (c1) 4% CS, 6% PVP, 0.2% GO - 10 mg/mL 5Fu | 577.20 | 132.5804 |
| (c2) 4% CS, 6% PVP - 10 mg/mL 5Fu | 1024.27 | 247.9765 |

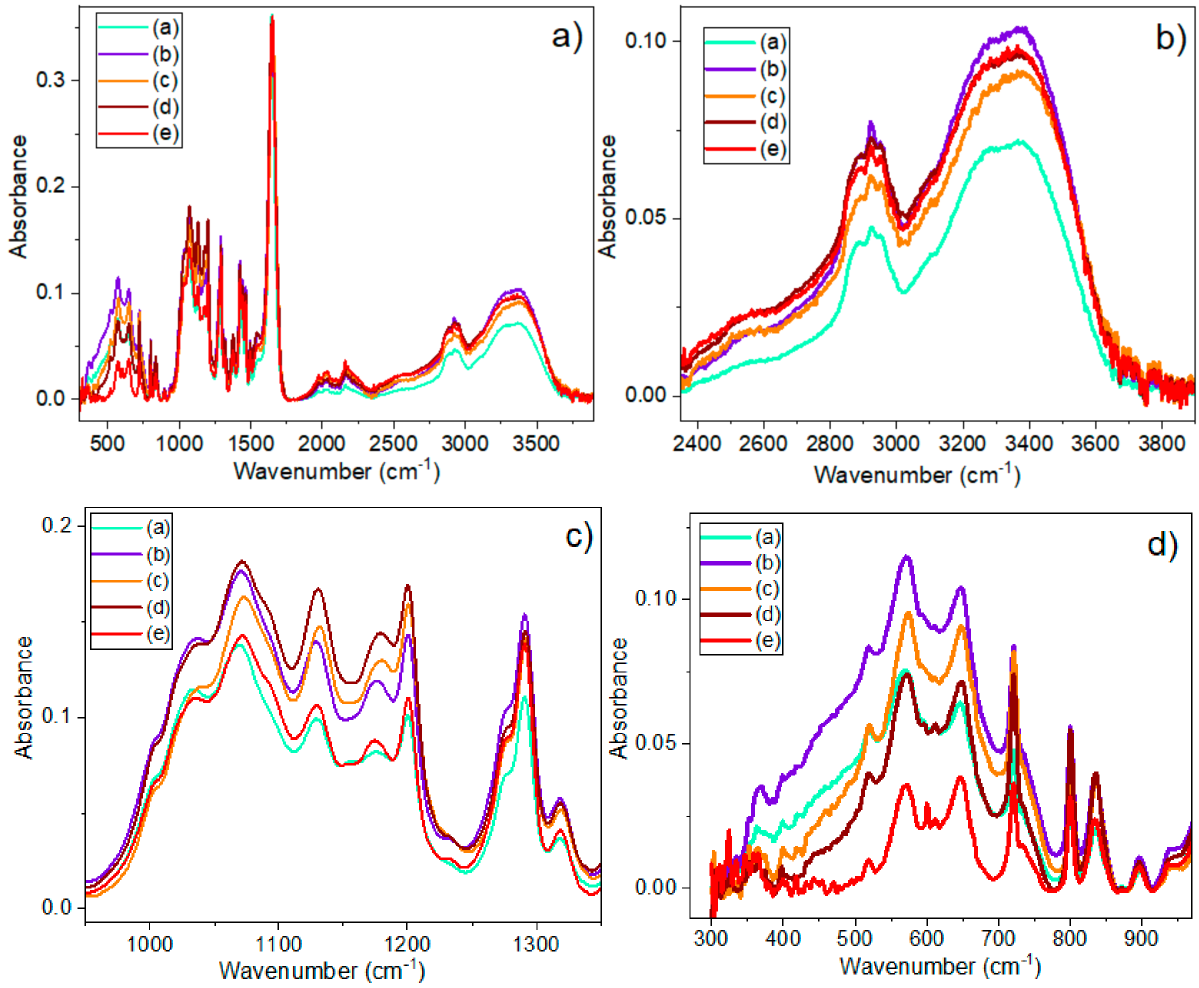


**Figure S2**. a) Infrared spectra of samples (a – e) shown in a) whole range of spectra, registered with FTIR-UATR, including detailed parts in b) high-frequency 2380-3900 cm$^{-1}$, c) intermediate 920-1350 cm$^{-1}$ and d) low-frequency 300-960 cm$^{-1}$ regions.

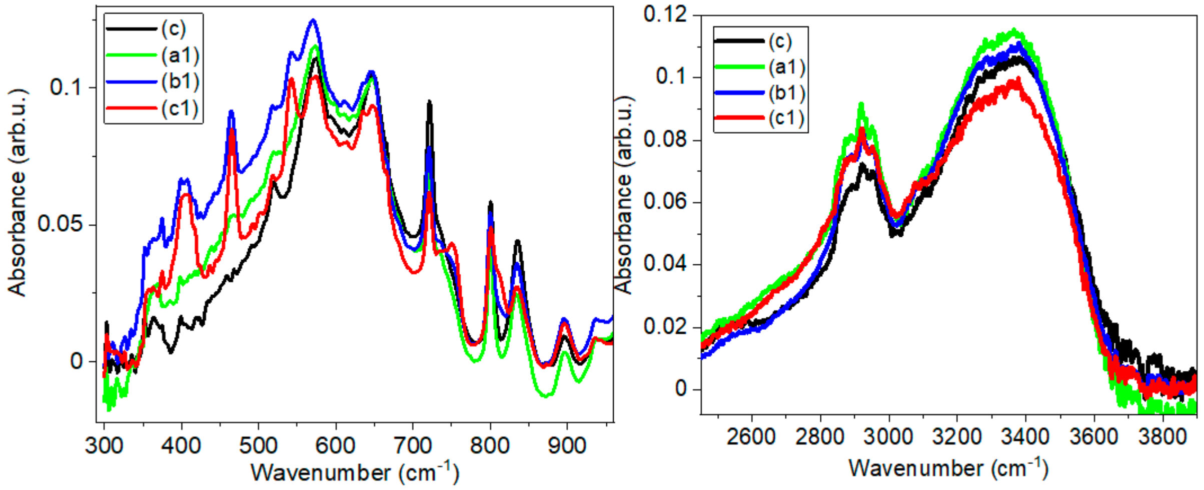


**Figure S3**. FTIR-UATR spectra of samples (c) (no 5-Fu), (a1) (1mg/ml of 5-Fu), (b1) (5 mg/ml 5-Fu) and (c1) (10 mg/ml 5-Fu) in a) low-frequency and b) high-frequency ranges of spectra.

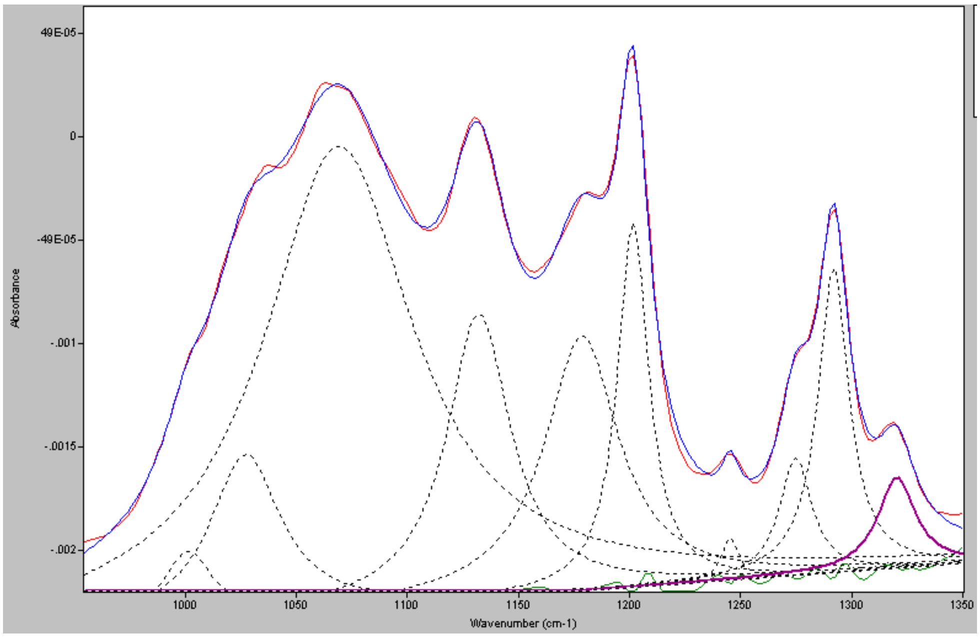


**Figure S4**. Example of fitting of FTIR spectrum of additional sample with 3 mg/ml 5-Fu in the region 960-1250 $cm^{-1}$.

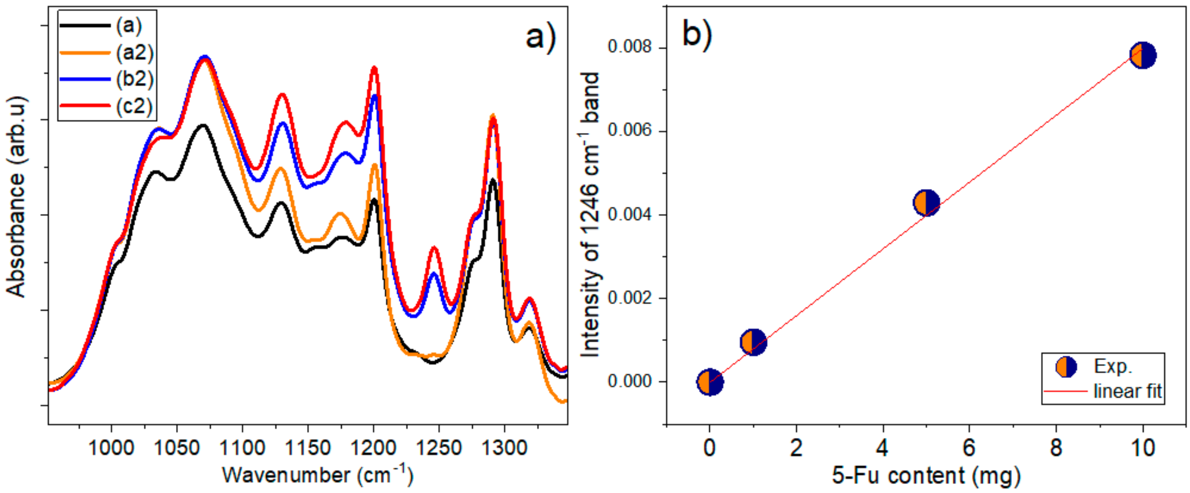


**Figure S5**. a) FTIR-UATR spectra of samples (a), (a2), (b2) and (c2), truncated in the region 964-1350 $cm^{-1}$. (b) The dependence of peak position of 1246 $cm^{-1}$ band vs 5-Fu content.

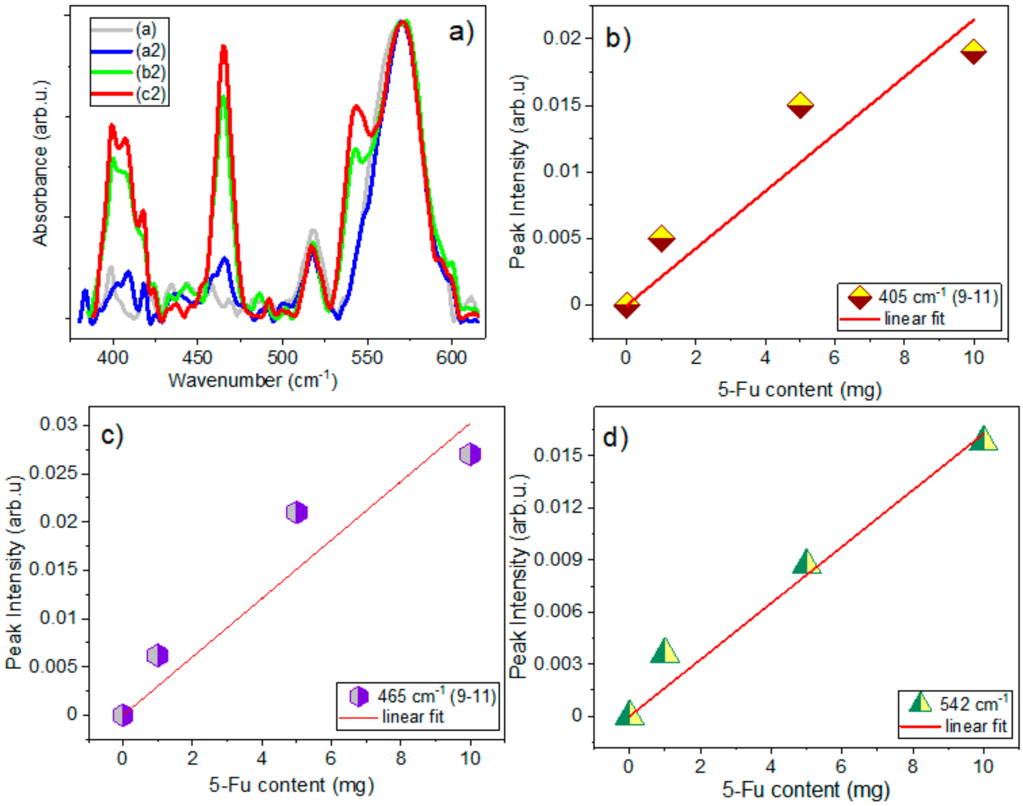


**Figure S6**. (a) Baselined spectral region of samples (a), (a2), (b2) and (c2), normalised to the intensity of 572 $cm^{-1}$ peak, for analysis of low-frequency vibrations. The dependence of peak position of low-frequency bands vs 5-Fu content, estimated from FTIR spectra shown in figure (a), for b) 405 $cm^{-1}$, c) 465 $cm^{-1}$ and d) 542 $cm^{-1}$ bands.

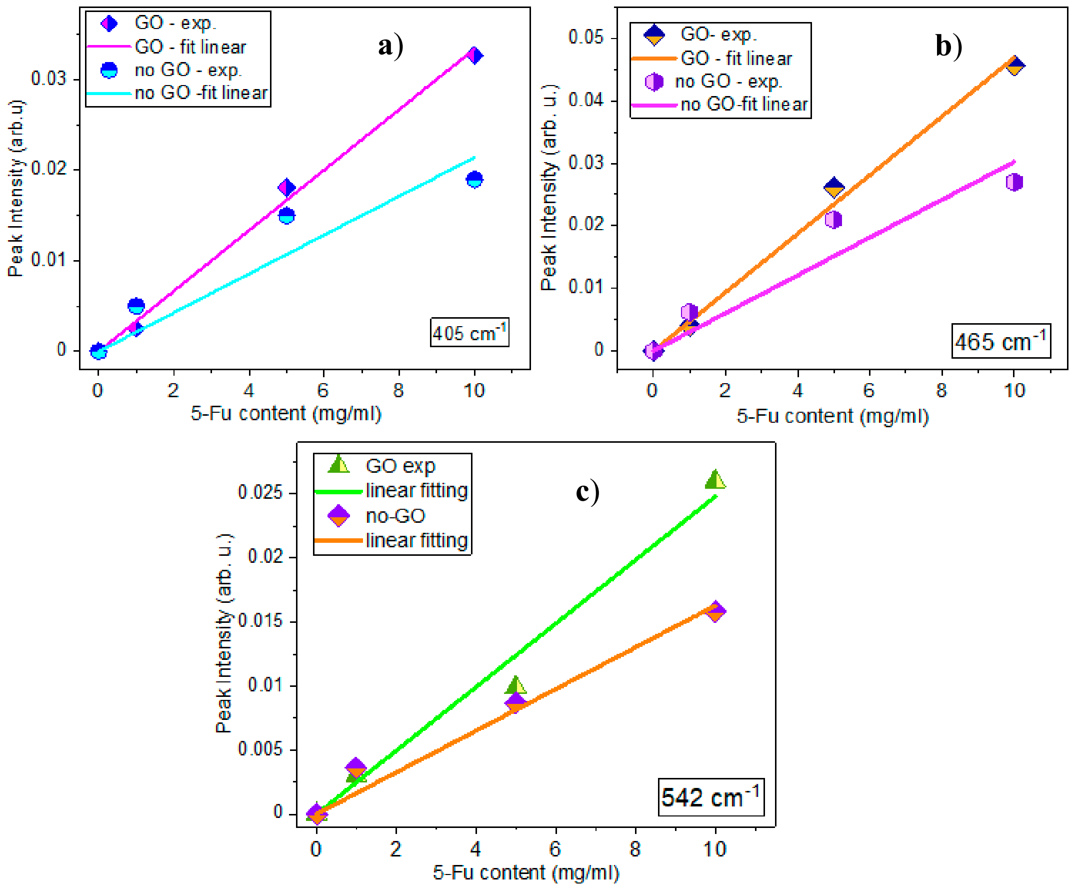


**Figure S7**. Comparison of dependencies of low-frequency IR peaks at a) 405, b) 465 and c) 542 cm$^{-1}$, related to 5-Fu, vs 5-Fu content for samples with 0.2% GO (c, a1, b1, c1) and without GO (a, a2, b2, c2). As follows from these graphs the slopes of the linear dependences for samples with GO is nearly 1.6 times larger than without GO.

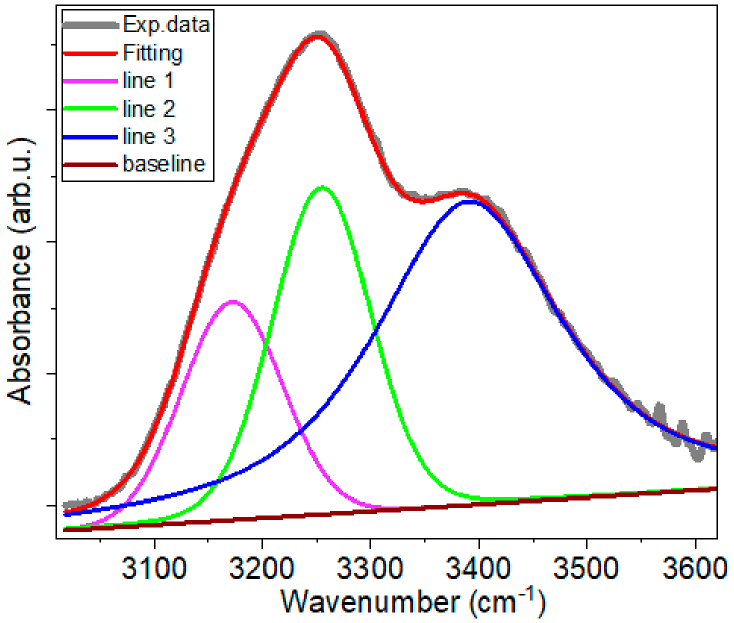


**Figures S8.** An example of fitting of one of FTIR spectra, shown in Figure 11a for sample (c) with 0% GO content, with 3 bands at around 3160, 3250 and 3387 cm$^{-1}$.

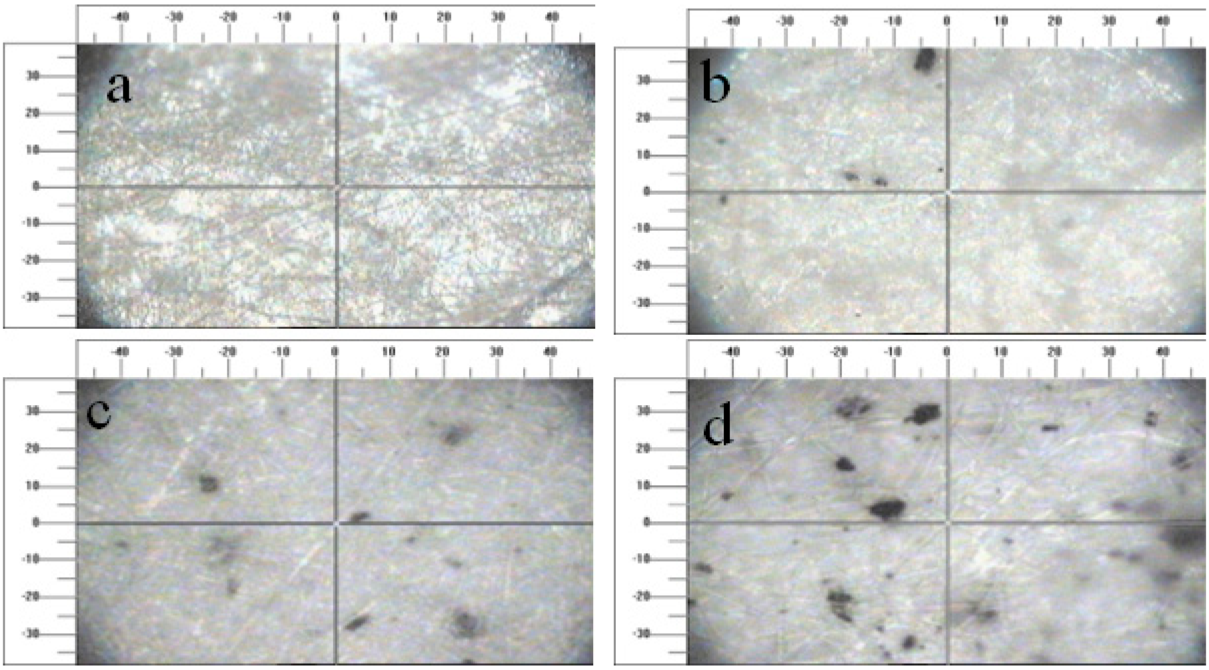


**Figures S9**. White light images registered on the Raman microscope of samples a) (a), b) (b), c) (d) and d) (e), which demonstrates the problems with focus onto fibres for samples (a) and (b) and easier to see fibres with a larger diameters for samples (d) and €. We can also see that the laser beam can be focused onto black spots which are a few microns in diameter, from which the registered spectrum may look differently with strong bands at around 1300 and 1600 $cm^{-1}$. The latter can be seen on example of Raman spectra shown in Figure S9.

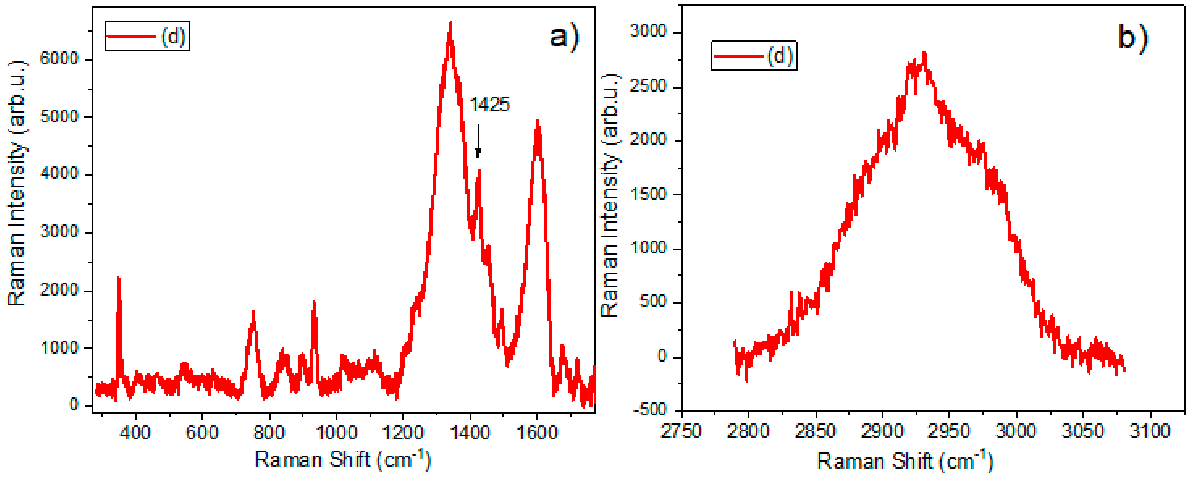


**Figure S10**. Raman spectra of sample (d) shown in low- a) and high- b) frequency sides, measured from the spot, shown in white-light image in Figure S9c).

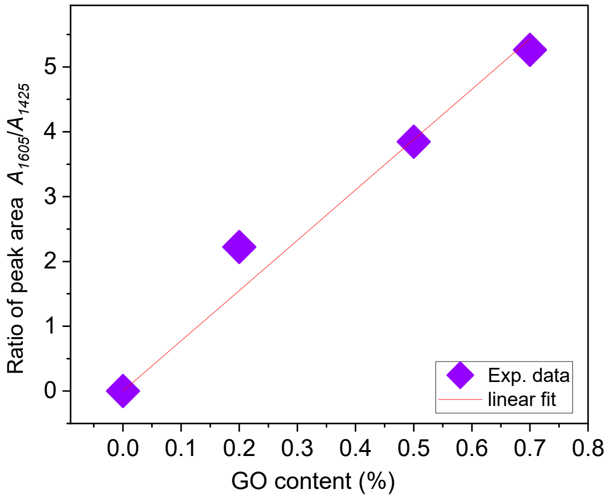


**Figure S11**. The ratio of integrated intensities $A_{1605}$ / $A_{1425}$ and vs GO content after fitting the Raman spectra for samples (a)-(e) in the region 1250 – 1750 $cm^{-1}$ with 6 bands.

**Table S4.** Tentative vibrational mode assignments for the normal Raman (NR) and SERS peaks for 5-fluorouracil (5-FU) and its metabolites 5-fluorouridine (5-FUrd), and 5-fluoro 2'deoxyuridine (5-FdUrd). From Ref. [55].

| 5-FU | | 5-FUrd | | 5-FdUrd | | Tentative Assignments |
|---|---|---|---|---|---|---|
| NR ($cm^{-1}$) | SERS ($cm^{-1}$) | NR ($cm^{-1}$) | SERS ($cm^{-1}$) | NR ($cm^{-1}$) | SERS ($cm^{-1}$) | |
| 365 | | 373 | | 369 | | Out-of-plane ring bend |
| 410 | | | | 418 | | In-plane ring bend |
| 467 | 488 | | 455 | 478 | 452 | In-plane ring bend |
| | | 492 | | 496 | | In-plane ring bend + ribose |
| 544 | | | | | | In-plane ring bend |
| | | | | 567 | | Out-of-plane ring and C=O bend |
| 635 | | | | | | In-plane ring bend |
| | | | 667 | 687 | 658 | Ring + ribose |
| | | | 717 | | | Ring + ribose |
| 732 | | 750 | | | | N-H out-of-plane bend |
| 766 | 786 | 767 | 794 | 764 | 791 | Pyrimidine ring breathing |
| 809 | 827 | 793 | 859 | 784 | 840 | Trigonal ring + C-F |
| | | 846 | | 860 | 896 | Ring + ribose |
| | | 892 | | 925 | | Ring + ribose |
| 933 | | | | | | Out-of-plane C6-H wag |
| 947 | | | | | | In-plane C6-H wag |
| | | | 954 | | | Ring + ribose |
| | | | | 1002 | | Ring + ribose |
| 994 | 1026 | | | | | Ring + N-H wag + C-H wag |
| 1183 | 1199 | | | | | N3-H in-plane bend |
| | | 1216 | 1205 | 1199 | 1209 | Ring + ribose |
| 1223 | 1234 | 1233 | 1236 | 1225 | 1242 | Ring + C-F |
| 1255 | 1275 | | | 1268 | | Ring str. |
| 1347 | 1334 | 1334 | 1339 | 1355 | 1345 | Ring + C-H wag |
| 1423 | 1400 | | 1388 | | 1395 | Ring + N-H wags |
| | | | 1457 | | 1450 | Ring str. + ribose $CH_2$ |
| 1502 | | | | | | N1-H in-plane bend |
| | 1545 | | | | | C6-H deformation |
| | 1606 | | 1602 | | | Trigonal ring mode |
| 1653 | | 1664 | | 1685 | | Ring C=C str. |
| 1669 | 1667 | 1695 | 1661 | 1713 | 1658 | Symmetric C=O str. |
| 1704 | | | | | | Anti-symmetric C=O str. |
| 1723 | | | | | | Fermi Resonance |

**Table S5.** From Ref. [44].

Comparison of the calculated harmonic vibrational wavenumbers (ω, $cm^{-1}$) in the dimer form of 5-fluorouracil at the B3LYP/6-31G** level, Relative infrared intensities (*A*, %), relative Raman scattering activities (*S*, %), scaled wavenumbers and experimental IR and Raman data ($cm^{-1}$) in the solid state of 5-FU.

| Calculated | | | Scaled[a] | Experimental IR | | Experimental Raman | | | No.[c] | Characterization |
|---|---|---|---|---|---|---|---|---|---|---|
| ω | *A* | *S* | | Ref. [27] | $\nu^b$ | SERS, Ref. [12]. | $\nu^b$ | Ref. [2] | | |
| 3659 | 9 | 37 | 3491 | **3141 vs** | 3135 | 3146 sh | | 3142.9 | 30 | ν(N1–H) |
| **3300**, 3258 | **100**, 0 | 0, 100 | **3152**, 3112 | **3069 vs** | 3067 | 3073 w | 3069 | 3066.5 | 29 | ν(N3–H) |
| 3243 | 0 | 28 | 3098 | **3001 vs** | 3020 | 3001 vw | | 3002.0, 3028.1 | 27 | ν(C6–H) |
| | | | | | | 2932,2892,2824 vw | | 2934.2, 2450.2, 2820.9 | | Combinations modes |
| 1851, **1849** | 0, **39** | 3, 0 | 1783, **1781** | **1722 s** | 1720 | 1725 vw, 1707 vw | 1706 | 1724.5, 1704.9 | 26 | ν(C2═O) + ν(C4═O) + δ(N–H) |
| **1773**, 1766 | **63**, 0 | 0, 22 | **1710**, 1703 | **1671 vs** | 1670 | 1672 m | 1671 | 1670.4 | 25 | ν(C4═O) + ν(C2═O) + δ(N3–H) |
| 1731, **1730** | 0, **3** | 9, 0 | 1670, **1669** | **1662 vs** | 1650 | 1659 w | 1655 | 1658.3 | 24 | ν(C5═C6) + ν(ring) |
| **1520**, 1519 | **4**, 0 | 0, 3 | **1471**, 1470 | **1502 m** | – | 1506 vw | 1504 | 1503.6 | 23 | δ(N1–H) + ν(ring) |
| **1480**, 1479 | **2**, 0 | 0, 9 | **1433**, 1432 | **1430 s** | 1435 | 1447 vw, 1425 w | 1425 | 1448.2, 1424.1 | 22 | ν(ring) + δ(N1–H) |
| 1429 | 4 | 1 | 1385 | **1349 m** | 1345 | 1350 vs | 1349 | 1348.5 | 20 | δ(N3–H) + δ(N1–H) + δ(ring) |
| | | | | **1312 w** | | 1313 vw | | 1311.3, 1119.3 | | Combinations modes |
| 1355, **1354** | 0, **2** | 11, 0 | 1315,**1314** | | 1260 | 1258 w | 1257 | 1256.8 | 21 | δ(C6–H) + v(ring) + δ(N1–H) |
| **1287**, 1286 | **18**, 0 | 0, 3 | **1250**, 1249 | **1225 m** | 1222 | 1225 m | 1226 | 1224.0 | 28 | ν(C–F) + v(ring) |
| 1219, **1218** | 0, **2** | 2, 0 | 1186, **1185** | **1181 m** | | 1187 vw | 1185 | 1186.8 | 18 | δ(C6–H) + δ(N3–H) + δ(ring) |
| 1169 | 3 | 1 | 1139 | **1158 sh** | 970 | 987 vw | 996 | 996.3, 985.5 | 19 | δ(CCH) + δ(N1–H) + δ(ring) |
| **979**, 975 | **3**, 0 | 0, 2 | **959**, 956 | **949 w** | | 950 vw | | 949.5 | 14 | δ(N3–H) + δ(C6–H) + δ(ring) |
| **937**, 908 | **9**, 0 | 0, 0 | **920**, 892 | | | 937 vw | | 936.0 | 9 | γ(N3–H) |
| **892**, 890 | **1**, 0 | 0, 1 | **877**, 875 | **880 m** | 875 | 887 vw | | 894.8 | 15 | γ(C6–H) |
| **823**, 820 | **3**, 0 | 0, 1 | **812**, 809 | **813 s** | | 810 vw | 811 | 809.1 | 17 | δ(ring) |
| 762, **755** | 0, **1** | 6, 0 | 754, **748** | **771 w** | | 779 sh, 769 m | 768 | 767.9 | 12 | δ(ring) |
| **748**, 747 | **1**, 0 | 0, 0 | **741**, 740 | **751 m** | 740 | 738 vw | | 734.7 | 11 | γ(C2═O) + γ(C4═O) + γ(ring) |
| 728 | 1 | 0 | 722 | **730 w** | | | | | 10 | γ(C–N3–H) + γ(ring) |
| **639**, 631 | **1**, 0 | 0, 2 | **638**, 631 | **643 m, 635 vw** | 615 | 639 w, 634 vw | 637 | 638.3, 633.5 | 7 | δ(ring) |
| **559**, 558 | **7**, 0 | 0, 0 | **563**, 562 | | | | | | 8 | γ(N1–H) |
| **541**, 539 | **2**, 0 | 0, 1 | **546**, 544 | **551 s, 517 w** | 546 | 546 w | 546 | 545.2 | 5 | 6b[d], δ(ring) |
| **464**, 462 | **1**, 0 | 0, 1 | **473**, 471 | **469 s** | 450 | 466 sh, 471 w | 470 | 470.0, 464 | 16 | δ(C–F) + δ(C═O) + δ(ring) |
| **408**, 398 | **3**, 0 | 0, 0 | **420**, 411 | **419** | 419 | 414 vw | 413 | 414 | 3 | δ(OCNCO) + δ(ring) |
| 388, **383** | 0, **1** | 1, 0 | 401, **396** | | | 399 sh, 366 vw | 367 | 365 | 4 | γ(C═C–H) + γ(ring) |
| 346 | 1 | 0 | 361 | | | 372 vw | | 371 | 13 | γ(NC═CF) + γ(ring) |
| 324, **321** | 0, **0.2** | 0, 0 | 341, **338** | | | 333 vw | 332 | 331 | 6 | δ(OCCF) + δ(ring) |
| 177, 165 | 0 | 0, 0 | 202, 190 | | | 207 vw | 210 | 207 | 1 | γ(C═O) + γ(N3–H) + γ(ring) |
| **117**, 116 | **0.2**, 0 | 0, 0 | **145**, 144 | | | 167 vw | 166 | 167 | 2 | γ(C═O) + γ(C–F) + γ(N1–H) + γ(ring) |
| 111,80 | 0 | 0, 1 | 139, 110 | | | 110 ms | 110 | 109 | | Lattice modes |
| 77,**65** | 0, **0.2** | 0, 0 | 107, **96** | | | 92, 83, 68 | | 91, 81, 76, 56 | | Lattice modes |
